# The adhesion-GPCR ADGRF5 fuels breast cancer progression by suppressing the MMP8-mediated antitumorigenic effects

**DOI:** 10.1038/s41419-024-06855-8

**Published:** 2024-06-27

**Authors:** Yalan Wu, Huixia Liu, Zhe Sun, Jieling Liu, Kai Li, Ronghui Fan, Fujun Dai, Hui Tang, Qi Hou, JinSong Li, Xiaolong Tang

**Affiliations:** 1 Department of Histology and Embryology, School of Basic Medical Sciences, Xiangya School of Medicine, Central South University, Changsha, 410013 China; 2 Hunan Key Laboratory of Animal Models and Molecular Medicine, School of Biomedical Sciences, Hunan University, Changsha, 410082 China; 3 Shanghai Key Laboratory of Regulatory Biology, Institute of Biomedical Sciences and School of Life Sciences, East China Normal University, Shanghai, 200241 China; 4 Department of Spine Surgery, The Third Xiangya Hospital of Central South University, Changsha, 410013 China; 5 Key Laboratory of Natural Medicine and Immuno-Engineering, Henan University, Kaifeng, 475004 Henan China; 6 Department of Neurosurgery, Nanchong Central Hospital, The Second Clinical Medical College, North Sichuan Medical College, Nanchong, 637003 Sichuan China; 7 Department of Urology, Shenzhen University General Hospital, Shenzhen University, Shenzhen, China; 8 International Cancer Center, Shenzhen Key Laboratory, Hematology Institution of Shenzhen University, Shenzhen, 518061 China

**Keywords:** Oncogenes, Breast cancer

## Abstract

ADGRF5 (GPR116) has been identified as a facilitator of breast cancer cell migration and metastasis, yet the underlying mechanisms remain largely elusive. Our current study reveals that the absence of ADGRF5 in breast cancer cells impairs extracellular matrix (ECM)-associated cell motility and impedes in vivo tumor growth. This correlates with heightened expression of matrix metalloproteinase 8 (MMP8), a well-characterized antitumorigenic MMP, and a shift in the polarization of tumor-associated neutrophils (TANs) towards the antitumor N1 phenotype in the tumor microenvironment (TME). Mechanistically, ADGRF5 inhibits ERK1/2 activity by enhancing RhoA activation, leading to decreased phosphorylation of C/EBPβ at Thr235, hindering its nuclear translocation and subsequent activation. Crucially, two C/EBPβ binding motifs essential for MMP8 transcription are identified within its promoter region. Consequently, ADGRF5 silencing fosters MMP8 expression and CXCL8 secretion, attracting increased infiltration of TANs; simultaneously, MMP8 plays a role in decorin cleavage, which leads to trapped-inactivation of TGF-β in the TME, thereby polarizing TANs towards the antitumor N1 neutrophil phenotype and mitigating TGF-β-enhanced cell motility in breast cancer. Our findings reveal a novel connection between ADGRF5, an adhesion G protein-coupled receptor, and the orchestration of the TME, which dictates malignancy progression. Overall, the data underscore ADGRF5 as a promising therapeutic target for breast cancer intervention.

## Introduction

Metastasis is the primary cause of mortality in breast cancer, wherein cancer cells colonize distant organs such as the lung, bone, liver, or brain [1]. Extracellular signals, including extracellular matrix (ECM) remodeling, growth factors, and cytokines in the tumor microenvironment (TME), play a crucial role in the development of malignant characteristics in cancer cells. In response to these extracellular stimuli—transmitting signals from the TME into cancer cells—various integral membrane proteins are involved, with G protein-coupled receptors (GPCRs) considered indispensable for processing a wide variety of TME signals. Significantly, about 50% of marketed pharmaceuticals target human GPCRs or their signaling pathways, underscoring their significant therapeutic potential [2]. ADGRF5 (GPR116) belongs to the family of adhesion G protein-coupled receptors (aGPCRs or ADGRs), the second-largest GPCR family containing 33 members. Most aGPCRs are orphan receptors with adhesion domains in their extracellular N-terminal regions, such as integrins, cadherins, and selectins, indicating their potential roles in communicating with the ECM [3]. Initially, ADGRF5 is found to have high expression in the lung [4] and plays critical roles in regulating lung surfactant and pulmonary alveolar homeostasis [5–10]. The loss of ADGRF5 in the lung causes emphysema-like symptoms by associating with alveolar macrophage activation [11] and airway inflammation induced by the expression of CCL2 in lung endothelial cells [12]. Moreover, recent studies have revealed that ADGRF5 inhibits renal acid secretion [13], mediates insulin-sensitizing effects [14], is involved in lipogenesis and fat browning [15, 16], maintains the skeletal muscle stem cell pool [17], regulates the pancreatic islet development [18], and prevents hepatic ferroptosis in liver injury [19]. Intriguingly, while the clinical relevance between ADGRF5 and various cancers is widely reported [20–28], the roles and underlying mechanisms of ADGRF5 in contributing to carcinogenesis and progression remain largely unknown. We previously have demonstrated that ADGRF5 promotes breast cancer metastasis via Gαq/11-mediated RhoA and Rac1 activation [29], highlighting its great potentials for prevention of breast cancer metastasis. Therefore, delving deeply into the mechanisms by which ADGRF5 dictates breast cancer progression is crucial for further clinical applications.

The matrix metalloproteinases (MMPs) family, representing the most prominent proteinases, is intimately associated with tumorigenesis by contributing to extracellular matrix turnover, cancer cell migration, cell growth, inflammation, angiogenesis, and remodeling of TME [30]. Interestingly, MMP8 stands out as one of the most distinct members with antitumorigenic and anti-metastatic functions. For instance, high MMP8 expression is linked to a significantly lower risk of cancer incidence and metastasis, as well as prolonged overall patient survival [31–35]. In terms of function, MMP8 has been found to inhibit breast cancer metastasis through the modulation of cell adhesion and invasion [36] and significantly inhibited cell growth both in vitro and in vivo through an unknown mechanism in melanoma [37]. Notably, MMP8 is also involved in the regulation of macrophage differentiation and polarization [38]. MMP8 has been reported to increase CXCL8 expression and suppress TGF-β signaling transduction through the trapping-inactivation of TGF-β in breast cancers [39, 40]. However, how MMP8 is regulated during cancer progression remains elusive.

Neutrophils, as a key component of the innate immune system, rapidly move to the forefront of defense against infections through processes such as phagocytosis, extracellular degranulation, and spreading of extracellular traps [41]. Recently, tumor-associated neutrophils (TANs) have been found to play distinct roles in tumor progression, responding to different stimuli released from the tumor microenvironment [42–44]. TANs were previously shown to have a pro-tumorigenic effect at the primary site by secreting pro-tumorigenic factors, promoting angiogenesis, and suppressing immune responses [45–47]. The pro-tumorigenic effects of neutrophils are TGF-β dependent, and upon TGF-β blockade, neutrophils were shown to switch from the pro-tumorigenic N2 phenotype to the antitumorigenic N1 phenotype [48]. Exploring novel mechanisms that modulate the polarization of TANs phenotype would be a promising strategy for cancer therapy.

In current study, we further investigated the roles of ADGRF5 in breast cancer malignant progression. Our results suggest that ADGRF5 influences TME education in breast cancers by transcriptionally suppressing MMP8 expression, increasing the potent utility of TGF-β in TME, and promoting the recruitment of TANs polarizing towards the pro-tumor N2 phenotype, thereby governing breast cancer progression. Our findings underscore ADGRF5 as a potential therapeutic target for breast cancers.

## Results

### Loss of ADGRF5 compromises malignant characteristics in breast cancers through the dysregulation of cell-extracellular matrix (ECM) and cell-cell interactions

**Fig. 1:**
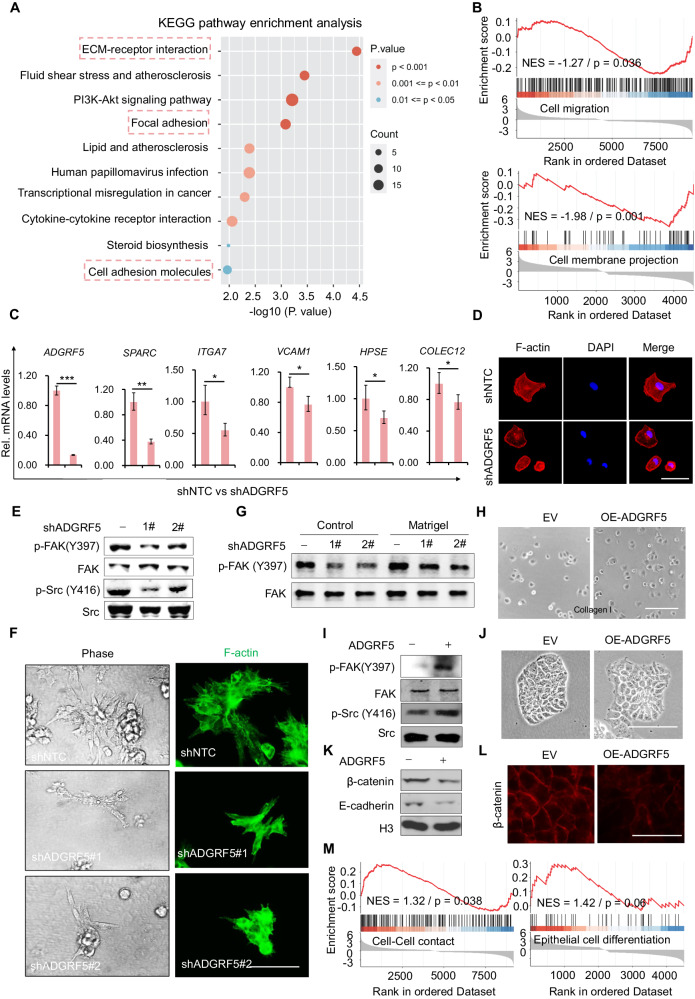
Loss of ADGRF5 compromises malignant characteristics in breast cancer through the dysregulation of cell-extracellular matrix (ECM) and cell-cell interactions. **A** KEGG analysis illustrating the changes in pathway enrichment based on microarray data in breast cancer MDA-MB-231 cells with ADGRF5 knockdown (shADGRF5) compared to control (shNTC) ones. **B** Gene set enrichment analysis (GSEA) revealing a negative enrichment of cell migration and cell-ECM interaction in shADGRF5 breast cancer cells. **C** qPCR analyzing the expression of genes associated with cell migration, cell-ECM, and cell-cell adhesion in MDA-MB-231 cells (n = 3 for each group) with or without ADGRF5 knockdown. **D** Fluorescence staining of F-actin polymerization by phalloidin in MDA-MB-231 cells with ADGRF5 knockdown or not. Scale bar, 50 μm. **E** Immunoblotting analysis of FAK phosphorylation at Y397 and Src at Y416 in MDA-MB-231 cells with or without silencing ADGRF5 expression. **F** Phase-contrast images (left) and fluorescence staining of F-actin polymerization (right) showing the morphology of shADGRF5 or shNTC MDA-MB-231 cells grown from the matrigel-embedded 3D culture model. Scale bar, 100 µm. **G** Immunoblots showing FAK phosphorylation at Y397 in breast cancer cells of (**F**). **H** Photos of MCF-7 cells with ADGRF5 overexpression or not seeded on collagen-coated culture dishes. Scale bar, 100 µm. **I** Immunoblotting analysis of FAK phosphorylation at Y397 and Src at Y416 in MCF-7 cells of (**H**). **J** Photos of cell colonies grown from MCF-7 cells with or without ADGRF5 overexpression. Scale bar, 100 µm. **K** Immunoblotting analysis of E-cadherin and β-catenin levels in cells of (**J**). **L** Immunofluorescence staining of β-catenin in MCF-7 cells with ADGRF5 overexpression or not. Scale bar, 50 μm. **M** GSEA analysis showing the positive enrichment of epithelial relevant cell-cell junction pathways in shADGRF5 cells. Data represent means ± SD in (**C**), and *p* values were calculated by two-tailed unpaired *t*-test. All representative results were collected from at least three independent experiments.

In our earlier study, we identified ADGRF5 as a promoter of breast cancer lung and bone metastasis through Gαq/11-mediated RhoA and Rac1 activation, indicating its potential as a promising target for breast cancer therapy [29]. Therefore, it is crucial to delve deeper into the functions governed by ADGRF5 and uncover the relevant mechanisms. To address this, we conducted the microarray-based whole gene expression analysis in MDA-MB-231 breast cancer cells with or without ADGRF5 knockdown. Interestingly, the loss of ADGRF5 (shADGRF5) resulted in significant expression changes in a large number of genes, particularly in pathways related to extracellular signaling transduction, such as ECM-receptor interaction, focal adhesion, and cell adhesion molecules (Fig. 1A, indicated by rectangles in dotted lines). Gene set enrichment analysis (GSEA) further revealed that ADGRF5 knockdown reduced cell motility, exemplified by negative correlation with cell adhesion and migration (Fig. 1B). To validate these findings, we investigated the expression of representative genes involved in breast cancer motility, including *SPARC*, *COLEC12*, *ITGA7*, *VCAM1*, and *HPSE*, found with significant downregulation in shADGRF5 cells (Fig. 1C). Among them SPARC promotes metastatic spreading in breast cancer [49], *VCAM1* is crucial for cancer cell extravasation and metastasis [50], and *ITGA7* knockdown inhibits cell invasion in breast cancer [51]. Given the significant impact on cell adhesion and ECM by ADGRF5 knockdown, we performed the phenotypic analysis of cell spreading, a core characteristic linked to cell adhesion, ECM-remodeling, and focal adhesion signaling [52, 53]. As shown, loss of ADGRF5 notably inhibited the cell spreading in MDA-MB-231 cells, resulting in a less elongated cell morphology and reduced polymerization of F-actin fibers (Fig. 1D and Supplementary Fig. 1A, B). Correspondingly, lower levels of FAK phosphorylation at Y397 and Src phosphorylation at Y416, both essential for efficient cell spreading [54], were evident in shADGRF5 cells (Fig. 1E and Supplementary Fig. 1C). In a more physiologically relevant 3D culture model by using matrigel [55], we found shADGRF5 cells formed smaller colonies with less cell protrusion at the leading edge, indicating a compromised malignancy phenotype compared to control cells appearing with elongated cell body and bridging multiple colonies [56] (Fig. 1F). Consistently, reduced FAK phosphorylation at Y397 was observed in 3D-cultured shADGRF5 cells (Fig. 1G and Supplementary Fig. 1D). Furthermore, ADGRF5 overexpression (OE-ADGRF5) in MCF-7 cells, which inherently express lower levels of ADGRF5 [29], markedly facilitated cell spreading (Fig. 1H) and increased phosphorylation of FAK at Y397 and Src at Y416, respectively (Fig. 1I and Supplementary Fig. 1E). OE-ADGRF5 cells grown colonies appeared irregular size, displayed more migratory cells at the colony edge, and exhibited looser cell-cell contacts compared to control cells expressing empty vector (EV) (Fig. 1J). In line with that, reduced E-Cadherin and β-catenin protein levels, two crucial components constituting epithelial junctions, were observed in OE-ADGRF5 cells (Fig. 1K, L), with their decline indicating potential EMT transition and increased breast cancer motility and malignancy [57]. Consistent with these observations, GSEA analysis indicated that loss of ADGRF5 enhanced cell-cell junctions, suggesting, at least partially, a reversal of the EMT process (Fig. 1M). Collectively, these results suggest that the loss of ADGRF5 impairs ECM-associated cell motility and inhibits breast cancer malignancy.

### ADGRF5 decline retards tumor growth and facilitates the polarization of TANs towards antitumor N1 neutrophils

**Fig. 2:**
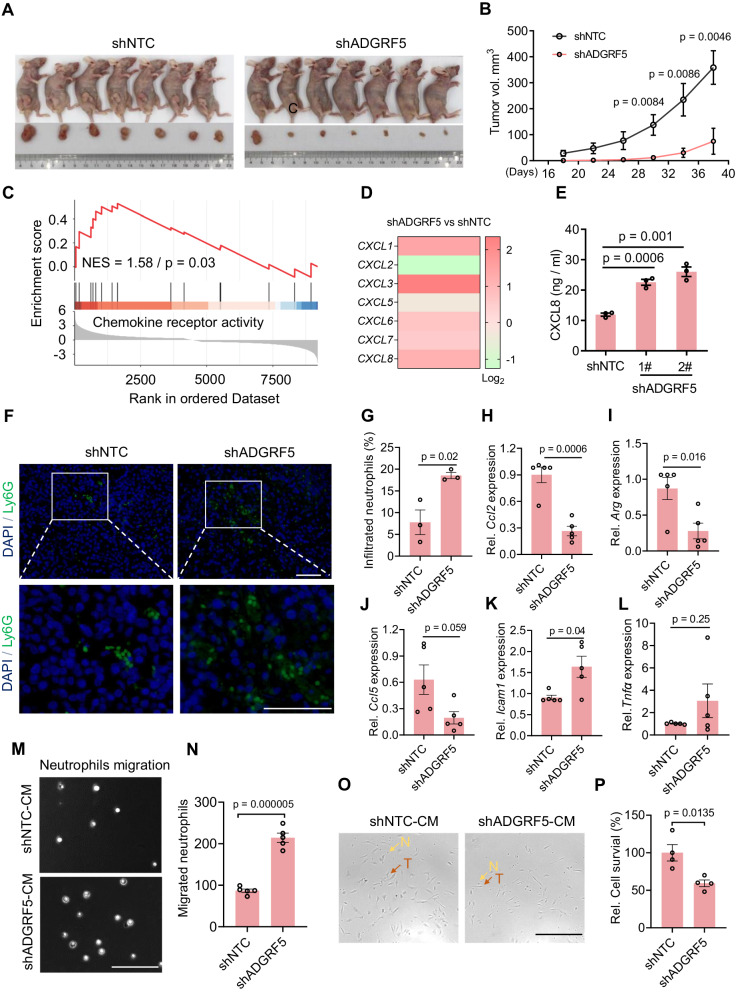
ADGRF5 decline retards tumor growth and facilitates polarization of TANs towards antitumor N1 neutrophils. **A**, **B** MDA-MB-231 cells stably expressing Scrambled (shNTC) and ADGRF5 knockdown shRNAs were injected into the mammary fat pad of mice (*n* = 7 for each group). Mice bearing tumors were sacrificed 38 days post-injection and shown in (A). Tumor size was measured using vernier calipers and tumor volume was calculated for generating the tumor growth curve in (**B**). **C** GSEA analysis showing the significant enrichment of chemokine receptor pathway in shADGRF5 breast cancer cells. **D** Heatmap representing the changes of chemokine genes contributing for neutrophil mobilization in shADGRF5 breast cancer cells. **E** CXCL8 secretion into the culture medium was detected by ELISA kit in MDA-MB-231 shADGRF5 and shNTC cells (*n* = 3 for each group). Immunofluorescence staining of Ly6G-marked neutrophils in the xenograft sections derived from shNTC and shADGRF5 breast cancer cells (**F**), and statistical analysis (*n* = 3 mice for each group) showing the proportion of infiltrated neutrophils (**G**). Scale bar, 100 µm. **H**–**L** qPCR analysis showing the mRNA changes of several essential genes indicating N1 neutrophils within xenografts derived from breast cancer cells with shNTC and shADGRF5 (*n* = 5 for each group). **M**, **N** Representative images (**M**) showing the neutrophils migrating through the transwell chamber, attracted by conditional medium (CM) obtained from shNTC and shADGRF5 breast cancer cells. The relative number of migrated cells (*n* = 3 for each group) was quantified in (N). Scale bar, 100 µm. **O**, **P** Representative images (**O**) showing the co-culture of normal MDA-MB-231 cells and neutrophils for 24 h with exposure to CM collected from shNTC and shADGRF5 MDA-MB-231 cells. The remaining surviving MDA-MB-231 cells was quantified in (**P**), n = 3 for each group. Red arrows indicate tumor cells (T); yellow arrows indicate neutrophils (N). Scale bar, 100 µm. All data represent means ± SEM, and *p* values were calculated by two-tailed unpaired *t*-test, collected from at least three independent experiments.

We then investigated the impact of ADGRF5 on breast cancer growth in vivo by injecting shADGRF5 and shNTC cells suspended in matrigel into the fourth mammary fat pad of nude mice. Results revealed that mice bearing shNTC cells developed significantly larger tumors than those receiving shADGRF5 cells 38 days post-injection (Fig. 2A, B). Interestingly, as per our previous study [29], ADGRF5 knockdown minimally affected cell growth in culture dishes, suggesting an influence on the tumor microenvironment (TME). Chemokine-directed modulation of the TME, which would affect specific immune cell trafficking, plays pivotal roles in cancer development, including tumor angiogenesis, cancer stemness, and metastasis [58]. As shown, a significant enrichment of chemokine pathway (Fig. 2C), accompanied by increased expression of several chemokines (CXCL1, CXCL3, CXCL4, CXCL5, CXCL6, CXCL7, and CXCL8) (Fig. 2D), was evidently observed in shADGRF5 cells. Profoundly, these chemokines are co-located within a narrow region of chromosome 4 and are reported to be co-regulated in malignant breast cancers, partially explaining the concurrent upregulation. Of great note, they belong to the CXC chemokine subfamily and possess potent chemotactic activity for neutrophils and proangiogenic properties [59, 60]. First of all, the tumor-suppressive effect observed with ADGRF5 knockdown contradicted the anticipated proangiogenic outcome, given the latter’s crucial role in tumor progression. In contrast, tumor-associated neutrophils (TANs) are known to exhibit both pro- and antitumorigenic effects within the TME, prompting our focus on these immune cells. A previous study compared the expression levels of these chemokines in ERα-negative breast tumors and in MDA-MB-231 cells used in our study, finding that CXCL8 had the most abundant expression [61]. Herein, although CXCL3 presented with the highest fold change in shADGRF5 cells (Fig. 2D, CXCL3 with 5.10 vs. CXCL8 with 2.22), its lower basal expression (CXCL3 at 4.44 vs. CXCL8 at 3396.69) [61] suggested a less significant impact compared to CXCL8 in our research model. Notably, CXCL8 is one of the most potent neutrophil-attracting chemokines, and its higher expression has been demonstrated to reduce tumorigenicity in immunodeficient mice through a neutrophil infiltration-dependent tumor-killing effect [44, 62]. Therefore, we focused on CXCL8 and its effect on TANs for further investigation. Consistently, secreted CXCL8 protein levels were higher in the supernatant of shADGRF5 cells (Fig. 2E); immunofluorescence staining of Ly6G, a neutrophil-specific marker [63], revealed a significant larger number of neutrophils infiltrating into shADGRF5 tumors compared to the shNTC controls (Fig. 2F, G). Additionally, macrophage infiltration showed marginal changes between tumors of shADGRF5 and shNTC (Supplementary Fig. 2A, B), emphasizing the prior contribution of neutrophils. The role of TANs in TME is characterized by plasticity and heterogeneity, particularly within the framework of the antitumor N1 phenotype and pro-tumor N2 phenotype paradigm [59]. To discern which type of TANs predominantly existed in shADGRF5 tumors, we examined gene profiles proposed to predict the shift of TANs phenotype [48]. Results indicated a significant decrease in the expression of *Ccl2*, *Ccl5*, and *Arginase* (Fig. 2H–J), but, to some extent, an increase in *Icam1* and *Tnfα* (Fig. 2K, L) in TANs of shADGRF5 tumors, suggesting the polarization of TANs towards antitumor N1 neutrophils. To provide further evidence, we isolated neutrophils from mouse bone marrow and tested the effect of ADGRF5 on TANs functions through in vitro assays. Firstly, the conditional medium (CM) collected from shADGRF5 breast cancer cells exhibited increased potency in attracting neutrophils migrating through transwell (Fig. 2M, emonstrated with higher efficiency in executing the tumor-killing function (Fig. 2O, P). Overall, these results suggest that the loss of ADGRF5 preventing breast cancer growth in nude mice was, at least partially, through switching the polarization of TANs towards antitumor N1 phenotype.

### ADGRF5 promotes the motility of breast cancer cells by suppressing the expression of antitumorigenic MMP8

**Fig. 3:**
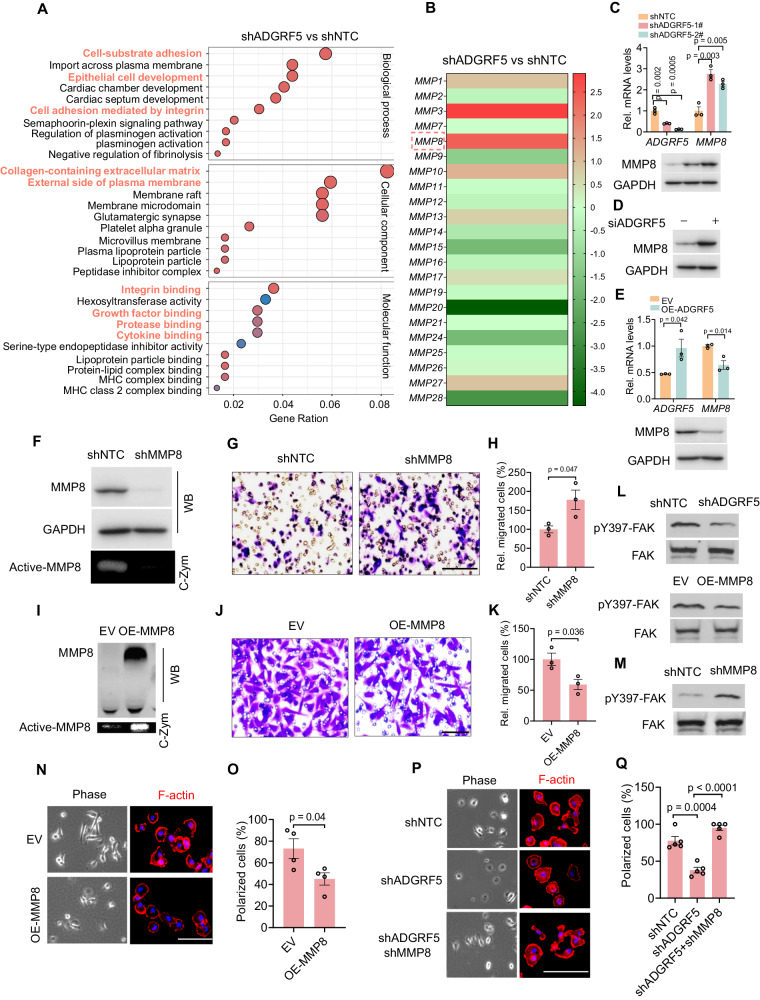
ADGRF5 promotes the motility of breast cancer cells by suppressing the expression of the antitumorigenic MMP8. **A** GO analysis revealing significant alterations in extracellular matrix (ECM) organization, cancer-associated ECM expression, and integrin binding (highlighted in pink) in shADGRF5 breast cancer cells. **B** Heatmap illustrating the expression changes of the matrix metalloproteinases (MMPs) between shADGRF5 and shNTC breast cancer cells. **C**–**E** qPCR and immunoblotting analyzing the *MMP8* mRNA and protein levels in breast cancer cells upon indicated treatments: MDA-MB-231 with ADGRF5 (**C**) and BT-549 cells (**D**) with ADGRF5 knockdown, and MCF-7 cells (**E**) with ADGRF5 overexpression. *n* = 3 for each group. **F** Immunoblots showing the MMP8 expression in MDA-MB-231 cells with indicated treatment, noting the MMP8 enzyme activity was detected by collagen zymography in the lowest panel (C-Zym). **G**, **H** Representative images (**G**) showing the transwell migration assay of MDA-MB-231 cells with MMP8 knockdown or not. The relative migration ability (*n* = 3 for each group) was calculated in (**H**). Scale bar, 100 µm. **I** Immunoblots showing the overexpression of MMP8 in MDA-MB-231 cells. The MMP8 enzymatic activity was examined by collagen zymography (C-Zym). **J**, **K** Representative images (**J**) showing the transwell migration assay in MDA-MB-231 cells with MMP8 overexpression or not. The relative migration ability (*n* = 3 for each group) was calculated in (**K**). Scale bar, 100 µm. **L**, **M)** Immunoblotting detecting the FAK phosphorylation at Y397 in cells with indicated treatments. **N**, **O** Representative images (**N**) displaying cell morphology and F-actin staining in MDA-MB-231 cells with MMP8 overexpression or not (N). Cell polarization proportion (*n* = 4 for each group) was counted in (**O**). Scale bars, 100 µm. **P**, **Q** Representative images (**P**) exhibiting cell morphology and F-actin staining in MDA-MB-231 cells with indicated knockdown of ADGRF5 or/and MMP8 expression (**P**). Cell polarization proportion (*n* = 5 for each group) was counted in (**Q**). Scale bars, 100 µm. All data are presented as means ± SEM, and *p* values were calculated by two-tailed unpaired *t*-test. Results were collected from at least three independent experiments.

We further aimed to elucidate the mechanisms by which ADGRF5 regulates breast cancer motility and TANs polarization. Given the profound impact on extracellular matrix (ECM) organization, cancer-associated ECM expression, and integrin binding observed in shADGRF5-altered cells (Fig. 3A, highlighted in pink), we focused on the matrix metalloproteinases (MMPs) family, known for its pivotal role in tumorigenesis by contributing to extracellular matrix turnover, cancer cell migration, cell growth, inflammation, angiogenesis, and the tumor microenvironment [30]. Our microarray data revealed significant downregulation of MMP9/20/24/28 and upregulation of MMP3/8 in shADGRF5 cells (Fig. 3B). Notably, MMP8, a reported tumor suppressor with high expression predicting a favorable prognosis in breast cancers [35–37, 40], stood out as the most impressive, although its underlying mechanism remained elusive. To investigate whether the antagonization of tumor malignancy induced by ADGRF5 knockdown was related to MMP8, we first corroborated the changes in MMP8 expression in MDA-MB-231 (Fig. 3C) and BT549 cells (Fig. 3D and Supplementary Fig. 3A) with ADGRF5 knockdown and in MCF-7 cells with ADGRF5 overexpression (Fig. 3E). Furthermore, restoring ADGRF5 expression significantly reversed the upregulation of MMP8 and CXCL8 induced by ADGRF5 knockdown, suggesting an ADGRF5-dependent effect (Supplementary Fig. 3B, C). Additionally, the negative correlation between ADGRF5 and MMP8 expression in breast cancer patients further reinforced these findings (Supplementary Fig. 3D). To assess whether MMP8 could be the key effector eliciting the observed changes in shADGRF5 cells, we generated MDA-MB-231 cells with stable knockdown or overexpression of MMP8 (shMMP8 or OE-MMP8) and related control cells (shNTC or EV) (Fig. 3F). As shown, transwell migration assays revealed a significant improvement in cell migration upon interfering MMP8 expression (Fig. 3G, H), while MMP8 overexpression resulted in evident inhibition (Fig. 3I–K). Similar to shADGRF5 cells, MMP8 overexpression impaired FAK phosphorylation at Y397 (Fig. 3L and Supplementary Fig. 3E), while MMP8 knockdown facilitated FAK phosphorylation (Fig. 3M and Supplementary Fig. 3F). Cell morphology analysis uncovered that MMP8 overexpression compromised cell spreading, characterized by insufficient cell body elongation and F-actin polymerization (Fig. 3N, O). Indeed, the ADGRF5 knockdown-conferred inhibition of cell spreading was largely reversed by interfering with MMP8 expression (Fig. 3P, Q). These findings collectively suggest that MMP8 is, at least partially, required for ADGRF5-mediated regulation of breast cancer motility.

### MMP8 underpins the polarization of TANs towards antitumor N1 neutrophils

**Fig. 4:**
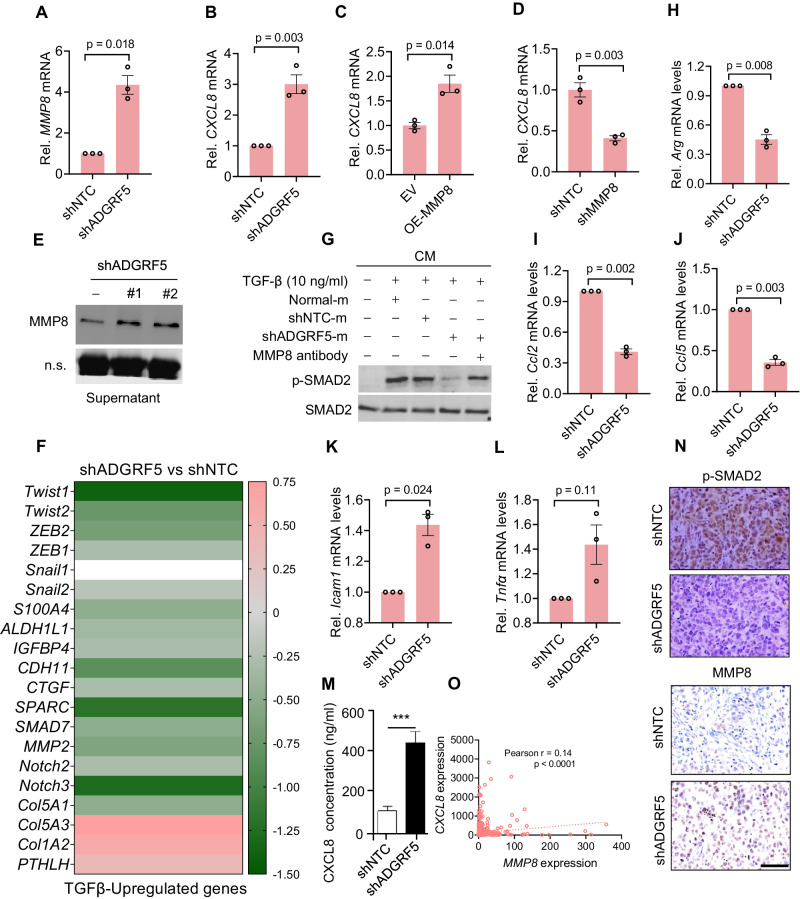
MMP8 underpins the polarization of TANs towards antitumor N1 neutrophils. **A**, **B** qPCR analysis of *MMP8* and *CXCL8* expression (*n* = 3 for each group) in MDA-MB-231 cells with ADGRF5 knockdown or not. qPCR analysis of *CXCL8* levels (*n* = 3 for each group) in MDA-MB-231 cells with MMP8 overexpression (**C**) or knockdown (**D**). **E** Immunoblots showing the secreted MMP8 protein in the cell culture medium derived from MDA-MB-231 cells with or without ADGRF5 knockdown. **F** Heatmap showing the expression changes of TGF-β-potentiated genes in breast cancer MDA-MB-231 cells with ADGRF5 knockdown or not. **G** Immunoblotting showing the phosphorylated SMAD2 in MDA-MB-231 cells exposed to conditional medium with indicated treatments. **H–L** qPCR analysis (*n* = 3 for each group) of server essential genes associated with N1 phenotype of TANs under the treatment of various conditional medium. **M** Bar chart showing the CXCL8 protein level (*n* = 3 for each group) examined by ELISA kit in the serum of mice bearing shNTC or shADGRF5 tumors. **N** Representative images showing IHC staining of the phosphorylation of SMAD2 (upper) and MMP8 protein expression (lower) in xenografts derived from MDA-MB-231 shADGRF5 cells and control cells. Scale bars, 100 μm. **O** The expression correlation analysis between *MMP8* and *CXCL8* mRNA in breast cancer patients. All data are presented as means ± SEM, and the *p* values were calculated by two-tailed unpaired *t*-test. Results were collected from at least three independent experiments.

We further investigated whether MMP8 played a role in education of TANs within the shADGRF5 tumors. Consistent with the simultaneous increase in *MMP8* and *CXCL8* expression by ADGRF5 knockdown in breast cancer cells (Fig. 4A, B), manipulation of MMP8 expression levels resulted in corresponding changes in in CXCL8 expression (Fig. 4C, D), suggesting that the increase in CXCL8 induced by ADGRF5 loss is, at least partially, dependent on MMP8, which aligns with a previous report indicating that MMP8 overexpression induced the upregulation of CXCL6 and CXCL8 in breast cancers [39]. While the increased neutrophil infiltration in shADGRF5 tumors can be attributed to MMP8-induced CXCL8 secretion, the mechanisms underlying the polarization of N1 TANs remained unclear. TGF-β is a key factor determining the pro-tumor N2 phenotype of TANs in the TME. Upon TGF-β blockade, TANs tend to polarize towards the antitumor N1 neutrophils [48, 59]. Notably, MMP8 has been reported to cleave decorin, which impairs the availability of TGF-β, i.e., the trapped-inactivation of TGF-β [40]. We thus asked whether the ADGRF5-MMP8 axis directly affected TGF-β signaling. Firstly, a significant increase in MMP8 secretion into the cell culture medium was observed (Fig. 4E), implicating a basis for modifying decorin in the TME. Secondly, based on previous microarray data, many genes potentiated by TGF-β signaling were noticeably decreased in shADGRF5 cells (Fig. 4F), including Twist1/2, ZEB1/2, Slug, and S100A4, which are known to respond to TGF-β activation [64], indicating the repression of TGF-β signaling by ADGRF5 knockdown. To assess the modulation of TGF-β availability by the ADGRF5-MMP8 axis in the TME, we used the conditional medium of shADGRF5 or shNTC cells preincubated with MMP8 antibody or normal IgG, with or without TGF-β supplementation, to treat MDA-MB-231 cells. The phosphorylation of SMAD2 was employed to evaluate the availability of TGF-β. Our results showed that cells treated with fresh culture medium (Normal-m) or shNTC-m presented much stronger SMAD2 phosphorylation than those with treatment of shADGRF5-m, while pre-incubation with the MMP8 antibody predicated to neutralize secreted MMP8, largely restored the phosphorylation level of SMAD2 (Fig. 4G and Supplementary Fig. 4A), suggesting that MMP8, at least partially, suppresses TGF-β signaling in shADGRF5 cells. Given these findings, we postulated that the ADGRF5-MMP8 axis would be beneficial for the polarization of antitumor N1 neutrophils. To test that, neutrophils isolated from mouse bone marrow were incubated with conditional medium (CM) of shADGRF5 or shNTC breast cancer cells. Examined by qPCR, several essential genes as aforementioned, including *Arginase 1*, *Ccl2*, and *Ccl5*, were significantly downregulated by CM of shADGRF5 cells (Fig. 4H–J), accompanied by some extent elevated levels of *Icam1* and *Tnfα* (Fig. 4K, L), reflecting the polarization of TANs towards antitumor N1 phenotype, as previously demonstrated [48]. To further consolidate our findings, relevant in vivo analysis was conducted. As shown, a significant increase of human-derived CXCL8 protein was detected in the serum of mice bearing shADGRF5 xenografts compared to the controlled ones (Fig. 4M). Additionally, as examined by IHC staining, the much lower phosphorylation of SMAD2 but higher MMP8 expression was presented in the shADGRF5 tumor sections (Fig. 4N). Moreover, the positive correlation between *MMP8* and *CXCL8* expression in breast cancer patients underlined their clinical relevance (Fig. 4O). In conclusion, our results demonstrate that ADGRF5 promotes breast cancer malignant progression, at least partially, by inhibiting antitumorigenic MMP8 expression. Loss of ADGRF5-induced MMP8 expression facilitates CXCL8 secretion and compromises TGF-β availability in the TME [40], thus leading to the polarization of TANs towards antitumor N1 neutrophils and disruption of cell motility and ECM-remodeling. On another front, considering the crucial roles of TGF-β in breast cancers, where it is highly active in malignant breast cancers and promotes metastasis [65], our findings underscore the potential mechanisms of ADGRF5-mediated G protein pathway synergizing with TGF-β signaling during breast cancer malignant progression.

### C/EBPβ is required for ADGRF5 loss-induced promotion of MMP8 expression in breast cancer cells

**Fig. 5:**
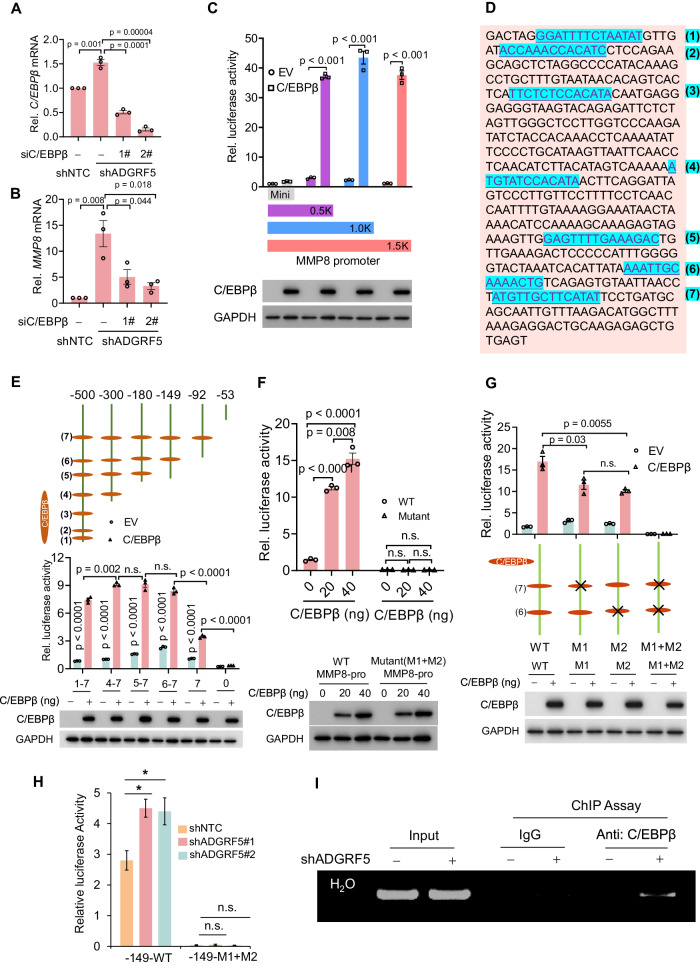
C/EBPβ is required for ADGRF5 loss-induced promotion of MMP8 expression in breast cancer cells. **A**, **B** qPCR analysis of *C/EBPβ* and *MMP8* mRNA levels (*n* = 3 for each group) in breast cancer MDA-MB-231 cells with or without C/EBPβ knockdown. **C** Luciferase reporter assay (upper) analysis of MMP8 promoter activity (*n* = 3 for each group) in HEK293T cells with C/EBPβ transfection or not. Immunoblots (lower) showing the expression of exogenous C/EBPβ. **D** Predicted C/EBPβ binding sites (highlighted in blue) in the MMP8 promoter using the online tool rVISTA 2.0. Numerals [1–7] indicated the potential binding motifs of C/EBPβ on the promoter region, started from −00 bp and ended at the transcription start site (TSS). **E** Luciferase reporter assay (upper) analysis (*n* = 3 for each group) showing the activity of MMP8 promoter truncations containing various C/EBPβ binding motifs. Immunoblots (lower) showing the expression levels of C/EBPβ transfected into HEK293T cells. **F** Luciferase reporter assay (upper) (*n* = 3 for each group) analyzing the activity of MMP8 promoter (−149 bp) containing the 6/7C/EBPβ binding motifs in HEK293T cells with dose-increased transfection of C/EBPβ. C/EBPβ expression was shown in the lower panel detected by immunoblotting. **G** Luciferase reporter assay (upper) analysis (*n* = 3 for each group) showing the activity of MMP8 promoter containing the 6/7 core C/EBPβ binding motifs (−149 bp, WT) and indicated mutant form (M1, 7-mutation; M2, 6-mutation; M1 + M2, combined mutation) in HEK293T cells with exogenous C/EBPβ transfection or not (lower). **H** Luciferase reporter assay (*n* = 3 for each group) showing the promoter activity of WT-MMP8 and the mutant form (M1 + M2) in breast cancer MDA-MB-231 cells with or without ADGRF5 knockdown. **I** C/EBPβ antibody and normal IgG-based ChIP-PCR showing the enrichment of C/EBPβ on the MMP8 promoter region in breast cancer cells with or without ADGRF5 knockdown. All data are presented as means ± SEM, and the *p* values were calculated by two-tailed unpaired *t*-test, collected from at least three independent experiments.

We used the rVISTA 2.0 online tool [66] to identify potential transcription factors (TFs) in the MMP8 promoter region. Notably, C/EBPβ, a canonical transcription factor, exhibited significantly higher scores, indicating its regulatory potential. To provide experimental validation, we employed oligo siRNAs to suppress C/EBPβ expression in MDA-MB-231 cells. Intriguingly, the elevated MMP8 expression resulting from ADGRF5 knockdown was markedly attenuated upon impairment of C/EBPβ expression (Fig. 5A, B). Further investigation, based on the analysis of MMP8 promoter region cloned into the PGL.417 luciferase vector, revealed that C/EBPβ-mediated MMP8 transcriptional activation was within the 0.5 kb promoter region (Fig. 5C), which contains seven conserved C/EBPβ binding motifs (Fig. 5D, highlighted in blue). Truncations of the MMP8 promoter region, including C/EBPβ binding motifs (-500bp/1–7, -300bp/4–7, -180bp/5-7, -149bp/6–7, and -92bp/7) or excluding them (−53 bp/0), were further cloned into the luciferase reporter system. C/EBPβ transfection increased luciferase activity without significant differences in constructs containing regions of −500 bp/1–7, −300 bp/4–7, −180 bp/5–7, and −149 bp/6–7, whereas a significant decline in activity was observed in the region of −92bp/7, and almost undetectable at −53 bp/0 (Fig. 5E), indicating the crucial role of the C/EBPβ binding motif at 149 bp/6–7. Indeed, the increased promoter activity of -149bp/6-7 was does-dependent response to C/EBPβ expression levels (Fig. 5F). To test binding specificity, we mutated the core C/EBPβ binding motif 5′TTGCA/T3′ (WT) to 5′CCGTT3′ (Mutant), as previously reported to impair C/EBPβ binding [67]. Luciferase reporter assays showed that mutation of the binding motif at either −149 bp/6 or −149 bp/7 significantly attenuated C/EBPβ-induced MMP8 promoter activity, while concomitant mutation achieved almost complete abolishment, indicating a synergistic effect of the two C/EBPβ motifs in potentiating *MMP8* transcription (Fig. 5G). In line with this, ADGRF5 knockdown in MDA-MB-231 cells increased WT-promoter activity but marginally affected the Mutant one (Fig. 5H). Finally, anti-C/EBPβ-based chromatin immunoprecipitation (ChIP) assays revealed prominently enhanced enrichment of C/EBPβ binding in the −149 bp/6–7 region in shADGRF5 cells (Fig. 5I). These findings collectively underscore the indispensable role of C/EBPβ in ADGRF5-mediated *MMP8* transcriptional regulation in breast cancer cells.

### ADGRF5 loss-induced MMP8 transcriptional activation is accomplished through ERK1/2-dependent phosphorylation of C/EBPβat Thr235

**Fig. 6:**
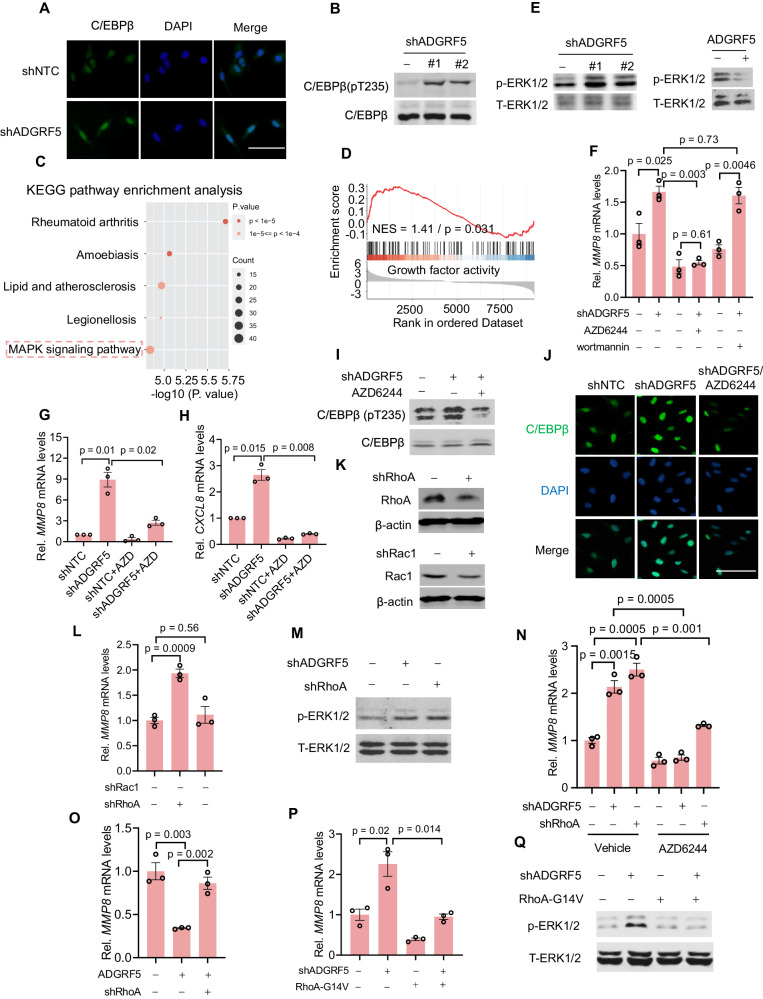
Impairment of ADGRF5-RhoA axis-induced MMP8 transcriptional activation is accomplished through ERK1/2-dependent C/EBPβ phosphorylation at Thr235. **A** Representative images showing the immunofluorescence staining of C/EBPβ in shNTC and shADGRF5 MDA-MB-231 cells. Scale bar, 100 μm. **B** Immunoblots showing the phosphorylation of C/EBPβ (pThr235) in MDA-MB-231 cells with or without ADGRF5 knockdown. KEGG pathway (**C**) and GSEA analysis (**D**) showing the enhanced activity of the ERK1/2 signaling pathway upon ADGRF5 knockdown in breast cancer cells. **E** Immunoblotting analysis of ERK1/2 phosphorylation levels in breast cancer MDA-MB-231 cells with ADGRF5 knockdown or MCF-7 cells with ADGRF5 overexpression. **F**–**H** qPCR analysis evaluating the mRNA expression levels of *MMP8* and *CXCL8* (*n* = 3 for each group) in breast cancer cells subjected to different treatments: ADGRF5 knockdown (shADGRF5), MEK inhibitor (AZD6244, 10 μM), and PI3K inhibitor (Wortmannin, 5 μM). **I** Immunoblots showing the phosphorylation of C/EBPβ at Thr235 in breast cancer cells with ADGRF5 knockdown and/or exposure to AZD6244. **J** Immunofluorescence staining of C/EBPβ in breast cancer MDA-MB-231 cells with indicated treatments. **K** Immunoblotting analysis of RhoA and Rac1 protein levels in MDA-MB-231 cells with expression of specific shRNAs. **L** qPCR analysis of the mRNA level of *MMP8* (n = 3 for each group) in breast cancer cells with knockdown of RhoA or Rac1. **M** Immunoblots showing the phosphorylation of ERK1/2 at Thr202 and Tyr204 in breast cancer cells with knockdown of ADGRF5 or RhoA, as indicated. **N** qPCR analysis of *MMP8* mRNA expression (*n* = 3 for each group) in breast cancer cells with RhoA or ADGRF5 knockdown, and exposed with or without the MEK inhibitor AZD6244. **O** qPCR analysis showing the *MMP8* mRNA levels (*n* = 3 for each group) in breast cancer MCF-7 cells with ADGRF5 overexpression or/and RhoA knockdown. **P**, **Q** qPCR and immunoblots analyzing *MMP8* mRNA levels (**P**) and ERK1/2 phosphorylation (**Q**) in shADGRF5 breast cancer cells with or without RhoA-G14V expression, *n* = 3 for each group). All data represent means ± SEM, and the *p* values were calculated by two-tailed unpaired *t*-test. Results were collected from at least three independent experiments.

C/EBPβ functions as a canonical transcription factor whose activity is strongly influenced by its nuclear translocation [68]. Intriguingly, as examined by immunofluorescence staining and immunoblotting, knockdown of ADGRF5 in breast cancer cells enhanced C/EBPβ nuclear translocation (Fig. 6A) and the phosphorylation at Thr235 (Fig. 6B and Supplementary Fig. 5A), suggesting its higher activity for transcription regulation. MAPK/ERK1/2 has been widely reported as a positive regulator of C/EBPβ activity via phosphorylating Thr235 [69]. Significantly, we found the evident upregulation of MAPK signaling pathway in shADGRF5 cells (Fig. 6C, D). Accordingly, the phosphorylation of ERK1/2, a key indicator of Ras/MAPK activation [70], was markedly increased in ADGRF5-knocked down MDA-MB-231 cells, but decreased in ADGRF5-overexpressed MCF-7 cells (Fig. 6E and Supplementary Fig. 5B, C). Furthermore, treatment with AZD6244, a kinase inhibitor of MEK [71], substantially attenuated the shADGRF5-induced increase in MMP8 expression, whereas PI3K inhibition by wortmannin [72] had a less profound effect (Fig. 6F). Indeed, AZD6244 simultaneously repressed the increase of CXCL8 expression elicited by loss of ADGRF5 (Fig. 6G, H). Moreover, the increased phosphorylation of C/EBPβ at Thr235 and promotion of nuclear translocation by ADGRF5 knockdown were apparently inhibited by AZD6244 (Fig. 6I, J and Supplementary Fig. 5D), demonstrating the direct regulation of ERK1/2 on C/EBPβ, consistent with a previous report [73]. Overall, these findings suggest that ADGRF5 loss-induced promotion of MMP8 expression occurs through the enhancement of ERK1/2-mediated C/EBPβ activation.

### ADGRF5-RhoA axis is pivotal for the regulation of ERK1/2 activity

We previously demonstrated that RhoA and Rac1 are two key effectors underlying the ADGRF5/Gαq signaling pathway, and the loss of ADGRF5 significantly suppresses the activation of RhoA and Rac1 [29]. We thus sought to investigate whether RhoA and/or Rac1 played a role in the regulation of MMP8 expression. To explore this, we initially used specific shRNA to interfere with the expression of RhoA and Rac1 in breast cancer MDA-MB-231 cells (Fig. 6K). Intriguingly, only the loss of RhoA markedly increased MMP8 expression, whereas Rac1 loss did not have a significant impact (Fig. 6L). Notably, both ADGRF5 and RhoA knockdown resulted in a noticeable increase in ERK1/2 activity (Fig. 6M and Supplementary Fig. 5E). Furthermore, the blockade of ERK1/2 largely abolished the RhoA knockdown-induced increase of MMP8 expression (Fig. 6N), and the inhibitory effect of ADGRF5 overexpression on MMP8 expression was significantly mitigated by interfering RhoA expression (Fig. 6O). We then asked whether RhoA-G14V, a constitutively active mutant form [74], could suppress MMP8 expression in shADGRF5 cells. As shown, the expression of RhoA-G14V significantly reversed ADGRF5 loss-induced MMP8 increase and ERK1/2 activation (Fig. 6P, Q and Supplementary Fig. 5F). Taken together, our results demonstrate that the ADGRF5/RhoA axis, as the upstream regulator, contributes to ERK1/2 inactivation, which suppresses C/EBPβ-dependent MMP8 transcriptional activation. This decline in MMP8 promotes breast cancer malignant progression by increasing TGF-β availability in the tumor microenvironment and promoting the polarization of tumor-associated neutrophils towards protumor N2 neutrophils. We thus highlight the significant therapeutic potential by targeting ADGRF5 for breast cancer treatment.

## Discussion

We previously reported that ADGRF5 promoted breast cancer metastasis through the Galphaq-p63RhoGEF-Rho GTPase pathway [29]. Our current study unveils more intricate roles of ADGRF5 in regulating breast cancer malignant progression. The ADGRF5-RhoA axis, a canonical G protein-coupled receptor signaling pathway, acts as the upstream regulator that suppresses ERK1/2 activity, necessary for the inhibition of C/EBPβ phosphorylation at Thr 235, thus contributing to the inhibition of antitumorigenic MMP8 expression in breast cancer cells. The MMP8 secreted by breast cancer cells into the tumor microenvironment (TME) cleaves decorin, leading to the trapped-inactivation of TGF-β, thereby disrupting TGF-β availability, by which it has a dual impact: compromising the protumorigenic effect of TGF-β on breast cancer cells, and promoting the polarization of TANs towards antitumor N1 phenotype. Consequently, the loss of ADGRF5 in breast cancer cells results in reduced cell motility, alterations in adhesion components, extracellular matrix (ECM) remodeling, and suppressed tumor growth. Hence, investigating the therapeutic efficacy of targeting ADGRF5 for breast cancer treatment is a promising avenue for future research.

ERK1/2 serves as a common effector for growth factor receptors, such as EGFRs [70]. Our data indicates that ADGRF5-mediated RhoA activation is, at least partially, if not entirely, responsible for the suppression of ERK1/2 activity. This finding might seem paradoxical in light of some reports suggesting that RhoA activates ERK1/2 [75]. However, studies have shown that the influence of RhoA on the nuclear localization of ERK1/2 is likely dependent on the cytoskeleton, and RhoA-associated actin disruption can lead to ERK1/2 activation, especially in MCF-7 breast cancer cells [76, 77]. Therefore, the observed suppression of ERK1/2 activity by ADGRF5 may be attributed to the significant impact of ADGRF5 on F-actin arrangement, as we have previously reported [29].

MMP8, a member of the MMPs family, is renowned for its role as a tumor-suppressive enzyme identified in various cancers [78–80]. However, limited studies have delved into the intricate mechanisms governing MMP8 expression. In our investigation, we pinpointed two crucial binding motifs on the MMP8 promoter for the transcription activator CCAAT/enhancer-binding protein β (C/EBPβ), demonstrating their indispensability for MMP8 transcriptional activation in breast cancer cells. Additionally, we uncovered that ADGRF5-mediated MMP8 regulation relied on C/EBPβ phosphorylation at Thr235 by ERK1/2, aligning with previous studies [81, 82]. Notably, PMA has been reported to induce MMP8 expression in diverse experimental models, though the precise mechanisms were previously unknown [83–85]. Our study provides plausible insights into the molecular basis for this phenomenon, considering the well-established downstream effects of PMA on the activation of ERK1/2 and C/EBPβ. Given the challenge of achieving specificity with MMP8 inhibitors for clinical translation, targeting upstream regulators like C/EBPβ and ERK1/2 may offer alternative strategies. Future research should explore the widespread existence of the ADGRF5-C/EBPβ-MMP8 axis in various tumor types.

The modulation of TME plays a crucial role in determining tumor progression [43, 86]. However, the dynamic orchestration of TME and the synergistic integration of complex signals during tumor development are still not fully understood. TANs are the important components of TME, acting as a double-edged sword in tumor progression similar to TAMs. The transition between the N1 (antitumor) and N2 (pro-tumor) phenotypes in TANs is reported to depend on TGF-β, enabling TANs to shift towards the N1 antitumorigenic phenotype upon blocking TGF-β [48]. In the current study, we observed that the inhibition of tumor growth resulting from ADGRF5 knockdown was, at least in part, determined by an enhancement in the polarization of TANs towards the N1 phenotype. Firstly, ADGRF5 loss-induced MMP8 expression promotes the secretion of CXCL8, the most potent chemotactic factor for neutrophil mobilization [87], leading to increased neutrophil infiltration into tumors. Secondly, due to MMP8-mediated cleavage of decorin, which in turn executes the trapped-inactivation of TGF-β in the TME [39, 40], infiltrated TANs exposed to such TME conditions with lower availability of TGF-β are more likely to maintain the antitumor N1 phenotype. It is noteworthy that TGF-β has been reported to suppress MMP8 expression during malignant progression in some cancers [88, 89], suggesting a potential strategy exploited by tumor cells to counteract the tumor-suppressive effect conferred by MMP8. In summary, our data emphasize that targeting ADGRF5 holds great potential for combating both breast cancer metastasis and growth.

## Materials and methods

### Cell culture

The human breast cancer cell lines mentioned in the manuscript were sourced from the American Type Culture Collection (ATCC^®^) and maintained at 37 °C with 5% CO_2_. All cell lines were routinely tested for mycoplasma contamination by PCR (TaKaRa, Japan). According to the recommended conditions, human breast cancer MDA-MB-231 and MCF-7, as well as HEK 293T cells were cultured in Dulbecco’s modified Eagle’s medium (DMEM, Sigma, D7777) supplemented with 10% fetal bovine serum (SenBeiJia Biological Technology, China). Breast cancer BT549 cells were cultured in RPMI 1640 medium (Thermo Fisher Scientific, No.23-400-021) with supplementation of 10% fetal bovine serum (SenBeiJia Biological Technology, China).

### Immunofluorescence staining

Following plating on coverslips and culturing in 24-well plates with the specified treatments, cells were fixed using 4% paraformaldehyde for 15 minutes, permeabilized with 0.1% Triton X-100/PBS buffer for 5 minutes at room temperature and blocked with 1% BSA for 30 min. Subsequently, the cells were incubated with the primary antibody at 4 °C overnight, followed by incubation with the appropriate secondary antibody for 1 hour at room temperature. Finally, immunofluorescence images were captured using a confocal laser scanning microscope (Leica TCS SP5). The antibody concentrations used were as follows: anti-C/EBPβ, 1 ug/ml; Alexa-488-conjugated anti-rabbit IgG, 1 ug/ml. For F-actin staining, cells underwent a similar treatment, but the addition of the primary antibody was replaced with phalloidin in PBS (1:500) for 30 minutes. All antibodies used in this study are listed in Supplementary Table 2.

### Transwell migration assay

For tumor cells, 4 × 10^4^ cells suspended in 200 µl FBS-free medium were placed in the upper well (8 µm pore size, Corning®). The insert was then incubated in a 24-well plate supplemented with 500 µl medium containing 10% FBS as the chemoattractant. After 6–12 h, as indicated in the manuscript, the assay was stopped. Then, the top membrane was swiped with cotton swabs to remove the non-migrated cells, and the migrated cells were stained with crystal violet before counting the cell number to evaluate the migration ability. For neutrophils, the pore size of the upper well was 3 µm, and the conditioned medium collected from tumor cells with the indicated treatment was employed as a chemoattractant. The number of migrated neutrophils was counted using a hemocytometer.

### RT-PCR, quantitative PCR (qPCR), siNRAs

Total RNA was isolated following the standard method from cell lysis in Trizol reagent (RNAiso Plus, Takara), and cDNA was synthesized using 5×Primescript® RT Master Mix (Takara), according to the manufacturer’s instructions. RT-PCR was performed using 2×Hieff® Ultra-Rapid HotStart PCR Master Mix (with Dye) (10157, Yeasen), and qPCR was carried out using SYBR Green Mix (Takara) and the relative gene expression was calculated according to the standard method. All primer sequences used in this study are provided in Supplementary Table 1.

### Cell transfection and stable cell line construction

To interfere with the expression of indicated genes, oligo siRNAs were synthesized by GenePharma Company (Shanghai) and transfected into cells using Lipofectamine®3000 reagent (Thermo Fisher), following the manufacturer’s instructions. All oligo siRNAs used in this study are provided in Supplementary Table 1. To construct stable cell line with overexpression or knockdown of gene expression, we generated generate lentivirus. For details, 10 µg of lentiviral construct containing the target gene or shRNA as indicated, along with 10 µg of pSPAX2 and 5 µg of pMD2G, were co-transfected into HEK 293T cells using polyethyleneimine (PEI). The supernatant containing lentivirus was collected 72 h after transfection and filtered through a 0.22 µm membrane (Sigma). Cells with infection of indicated lentivirus were then selected by puromycin (MCE, HY-B1743A) or sorted by flow cytometry (FACS). All oligo sequences used in this study are provided in Supplementary Table 1.

### Microarray-based gene expression analysis

Microarray-based gene expression analysis was conducted to investigate alterations in gene expression within breast cancer MDA-MB-231 cells with ADGRF5 knockdown or not. The Affymetrix GeneChip® gene expression analysis array (Hum U133 plus 2.0) was utilized following the manufacturer’s instructions. Bioinformatic analysis for gene pathway enrichment was performed using the R package, adhering to standard protocols. The microarray data of this study are provided in Supplementary Table 3.

### Immunoblotting and antibody

Samples for immunoblotting were prepared in laemmli buffer and then subjected to SDS-polyacrylamide gel electrophoresis. Separated proteins were transferred to PVDF membranes (Millipore). After being blocked for 1 h in 5% non-fat milk-TBST (w/v), the membrane was incubated with the indicated primary antibody at 4 °C overnight. After being washed three times with TBST, immunoblotting images were captured using the Baygene detection system (BG-gdsAUTO 710 MINI) after incubation with the indicated HRP-conjugated secondary antibodies and proper washing. Antibodies against ERK1/2, Phospho-ERK1/2, SMAD2, Phospho-SMAD2, Phospho-C/EBPβ, and C/EBPβ were procured from Cell Signaling Technology. Monoclonal anti-β-actin, anti-GAPDH, and anti-Tubulin antibodies were obtained from Beyotime, while anti-Flag and anti-Rac1 antibodies were sourced from Sigma-Aldrich. The anti-MMP8 antibody was purchased from Santa Cruz Biotechnology. Detailed information regarding antibody applications and dilutions can be found in Supplementary Table 2.

### Conditional medium (CM) collection

Human breast cancer cells were initially cultured in the normal medium for growth. Once the cells reached 40% to 60% confluence, the cultured medium was replaced by fresh DMEM with the indicated FBS concentration. After 48 h, this medium was collected as conditional medium and stored at −80 °C for later use.

### Xenograft model of breast cancer in mice

All animal study was conducted following the accepted standards of the Ethics Committee of Hunan University. The orthotopic injection of breast cancer cells into the mammary fat pad was performed as previously described [90]. For details, pathogen-free female BALB/c mice at the age of 5–6 weeks were purchased from the Hunan SJA Laboratory Animal Co., Ltd (Changsha). Breast cancer MDA-MB-231 cells (1 × 10^6^) suspended in matrigel were injected into the fourth mammary fat pad of nude mice (*n* = 7 mice per group). Tumor diameters were measured and the tumor volume (mm^3^) was calculated according to the formula: volume = 0.5 × length × width^2^. In this study, no statistical method was used to determine the sample size. Mice were randomly incorporated into experimental groups and experiments were not performed in a blind manner. No mice were excluded in this study.

### Immunohistochemistry (IHC)

For IHC staining of tumor sections, tumors fixed in paraformaldehyde were embedded in paraffin and sectioned. Paraffinized sections were treated with 0.3% hydrogen peroxide/methanol, followed by incubation with antibodies diluted in Immunol Staining Primary Antibody Dilution Buffer (Beyotime, #P0103). Subsequently, sections were incubated with reagents from the Histostain-Plus IHC Kit (Rabbit Primary, Mt-bio#LHK612) following the manufacturer’s instructions. Detailed information regarding antibody applications and dilutions can be found in Supplementary Table 2.

### Purification of mouse bone marrow neutrophil

Eight- to twelve-week-old C57BL/6 male mice were euthanized, and femurs and tibias from both legs were dissected. The ends of each femur and tibia were clipped with sterile dissecting scissors. Bone marrow cells were flushed with HBSS without calcium and magnesium, and the solution was filtered through a 70 μm nylon cell strainer. Cells were collected by centrifuging at 400 × *g* for 5 min at 4 °C with low brake, and cell pellets were resuspended in red blood cell lysis buffer (RCLB, 0.15 M NH4Cl, 10 mM KHCO3, and 0.1 mM Na2EDTA, Sigma-Aldrich) to remove erythrocytes. Neutrophils were isolated using a three-layer Percoll gradient of 78%, 69%, and 52%, and collected between the 78% and 69% cell layers. After isolation, neutrophils were resuspended in serum-free DMEM for further procedures. The animal study was conducted following the accepted standards of the Ethics Committee of Hunan University.

### Statistical analyses

Statistical analysis was conducted using GraphPad® Prism 8 or Microsoft Excel. For in vitro experiments, representative results were collected from at least three independent experiments. The data are presented as the means ± standard error of the mean (SEM) or the mean ± standard deviation (SD) as indicated in the figure legend. The two-tailed unpaired *t*-test was performed for comparison between two groups and significance is defined as *p* < 0.05.

### Supplementary information

Supporting Information

Supplementary Material_Supplementary Table 3

Original western blots

## Data Availability

The gene expression correlation analysis of cancer patients is based on the public online tool TCGA. The other data that support the findings of this study are available from the corresponding author upon reasonable request.
