# Supplementary material for: The adhesion-GPCR ADGRF5 fuels breast cancer progression by suppressing the MMP8-mediated antitumorigenic effects
Source: Cell Death Dis. 2024 Jun 27;15(6):455. doi: 10.1038/s41419-024-06855-8 (PMC11211477; doi:10.1038/s41419-024-06855-8)
Supplement: Supplementary file 1 — Supporting Information [file 41419_2024_6855_MOESM1_ESM.pdf]

Supporting Information

This file contains Supplementary Figures 1-5 and Supplementary Tables 1 and 2.

Note: Microarray data in Supplementary Table 3 is uploaded as a separate file.

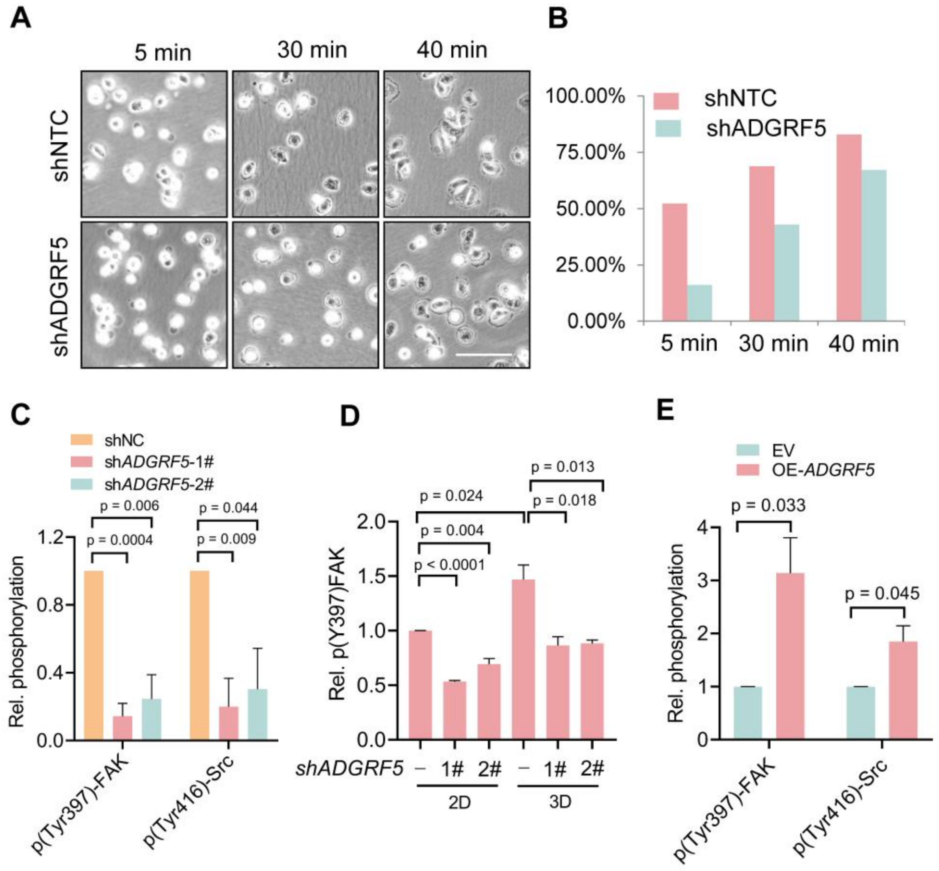

Supplementary Figure 1. Loss of ADGRF5 compromises cell motility in breast cancer cells

(A-B) Phase-contrast images (A) depicting the cell morphology of shADGRF5 and shNTC MDA-MB-231 cells post seeding at specified time points (5 minutes, 30 minutes, and 40 minutes), noting the bar chart (B) for the quantification of well-spreading cells which displayed elongated cell body. **(C-E)** Bar charts represented the quantification of protein phosphorylation levels related to Figure 1E, 1G, and 1I, respectively, n = 3 for each group. Data are presented as means ± SEM, and p-values were calculated by two-tailed unpaired *t*-test. The representative results were collected from at least three independent experiments.

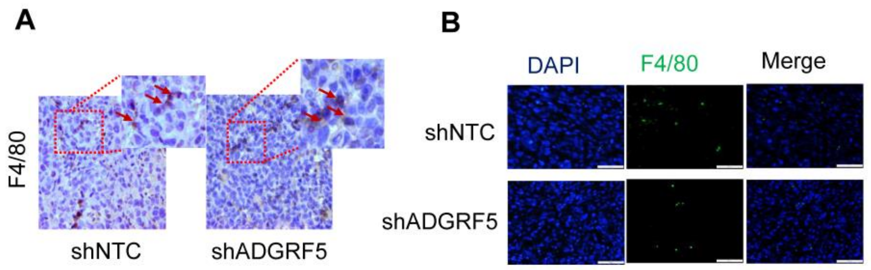

Supplementary Figure 2. Loss of ADGRF5 has marginal effect on the infiltration of macrophages into the TME

(A-B) Immunohistochemical (IHC) and immunofluorescence (IF) staining of the macrophage infiltration by using the F4/80 antibody in the tumor sections of shADGRF5 and shNTC.

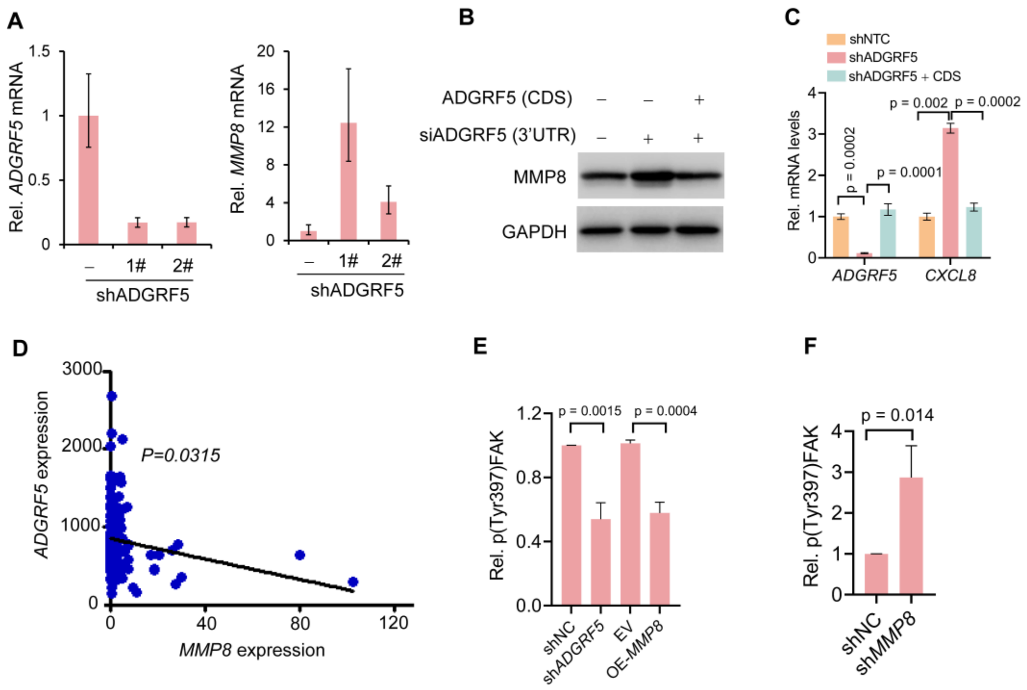

Supplementary Figure 3. MMP8 is essential for ADGRF5 knockdown-conferred cell motility impairment

(A) qPCR analysis of the mRNA levels of *ADGRF5* and *MMP8* (n = 3 for each group) in breast cancer BT549 cells with or without knockdown of ADGRF5. **(B-C)** Immunoblotting (B) and qPCR (C) showing the expression of MMP8 and CXCL8 in shADGRF5 MDA-MB-231 cells with the transfection of exogenous CDS region of ADGRF5, n = 3 for each group. **(D)** Correlation analysis depicting the relationship

between *ADGRF5* and *MMP8* mRNA expression in breast cancer patients. **(E-F)** Bar charts represented the quantification of protein phosphorylation levels related to Figure 3L and 3M, n = 3 for each group. All data represent means ± SEM, and p-values were calculated by two-tailed unpaired *t*-test, collected from at least three independent experiments.

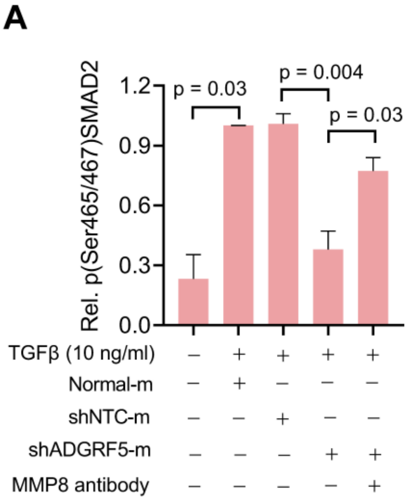

Supplementary Figure 4. ADGRF5 loss-conferred TGFβ signaling inhibition is MMP8 dependent

(A) Bar charts represented the quantification of SMAD2 phosphorylation levels related to Figure 4G, n = 3 for each group. Data are presented as means ± SEM, and p-values were calculated by two-tailed unpaired *t*-test. Results were collected from at least three independent experiments.

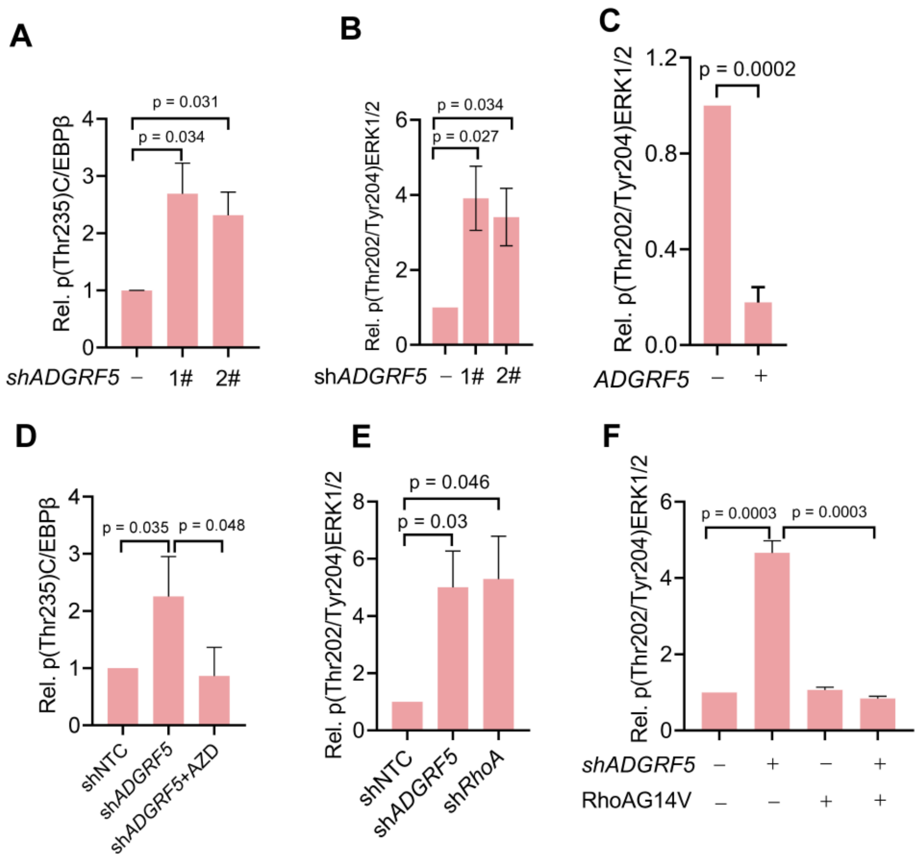

Supplementary Figure 5. ADGRF5-RhoA axis regulates MMP8 expression through the modulation of ERK1/2-C/EBPβ activity

(A-E) Bar charts represented the quantification of protein phosphorylation levels related to Figure 6B, 6E, 6I, 6M, and 6Q, respectively, n = 3 for each group. All data are presented as means ± SEM, and p-values were calculated by two-tailed unpaired *t*-test. Results were collected from at least three independent experiments.

Supplementary Table 1. Oligonucleotides used in this study

Genes	Forward (5'-3')	Reverse (5'-3')	Purpose
h*C/EBPβ*	ATCGGTTTAAACATG GCTGA	AGACGCCTCTTTTCT CATAG	qPCR
h*MMP8*	AGTGGGAACGCACTA ACTTG	TGAGCATCTCCTCCA ATACC	qPCR
h*CEBPβ*	TTGTCCAAACCAACC GCACA	GTTACACGTGGGTTG CGTCA	qPCR
h*β-actin*	AGCGAGCATCCCCCA AAGTT	GGGCACGAAGGCTC ATCATT	qPCR
m*β-actin*	CGTTGACATCCGTAA AGACC	AACAGTCCGCCTAGA AGCAC	qPCR
h*COLEC12*	TACTACAAGGTCCAC CGGG	TTAGATCCTTTGCCG CCACG	qPCR
h*HPSE*	TAGAGCTCTCGACTC TCCGCT	CGAAGCTTTGGAGAA CCCAG	qPCR
h*SPARC*	TTGACGGGTACCTCT CCCAC	ATTGGGGGAAACAC GAAGGG	qPCR
h*ITGA7*	GGCGTGCGAGATTTC CCTT	TGCATATCAGCTCCC TGGTC	qPCR
h*VCAM1*	AAATGCCTGGGAAG ATGGTCG	AGGAAAAGAGCCTG TGGTGC	qPCR
h*MMP9*	CGATGACGAGTTGTG GTCCC	TGTAGAGTCTCTCGC TGGGG	qPCR
h*IL-11*	AGCTGCAAGGTCAAG ATG	CCAAAGTGCCAGGAT TAC	qPCR
h*CXCL8*	TTGGCAGCCTTCCTG ATTTC	AACTTCTCCACAACC CTCTG	qPCR
m*Tnfα*	ACCACGCTCTTCTGT CTACT	AGGAGGTTGACTTTC TCCTG	qPCR
m*Vcam1*	GAAGCTTCTTTTGCT CTGCC	AGCAGTACTGGCACC AGAAT	qPCR
m*Arginase*	GATTGGCAAGGTGAT GGAAG	TCAGTCCCTGGCTTA TGGTT	qPCR
m*Ccl2*	CAGGTCCCTGTCATG CTTCT	GTCAGCACAGACCTC TCTCT	qPCR
m*Ccl5*	ACCATGAAGATCTCT GCAGC	TGAACCCACTTCTTC TCTGG	Knockdown
si*MMP8*	GCCUUGAUGUAUCC CAACUAU	AUAGTTGGGAUACA UCAAGGC	Knockdown

sh*RhoA*	CCGGGAAAGCAGGTAGAGTTGGCTTCTCGAGCTTTCGTCCATCTCAACCGAATTTTTG	5AATTCAAAAAGAAAGCAGGTAGAGTTGGCTTCTCGAGCTTTCGTCCATCTCAACCGAA	Knockdown
sh*Rac1*	CCGGCGCAAACAGATGTGTTCTTAACTCGAGTTAAGAACACATCTGTTTGCGTTTTTG	AATTCAAAAACGCAAACAGATGTGTTCTTAACTCGAGTTAAGAACACATCTGTTTGCG	Knockdown
si-h*CEBPβ*	GGCCCUGAGUAAUCGCUUA	UAAGCGAUUACUCAGGGCC	Knockdown
si-h*CEBPα*	GGCCCUGAGUAAUCGCUUA	UAAGCGAUUACUCAGGGCC	Knockdown
si*ADGRF5*-1	CCUUGUGUUCCAUAUCAUA	UAUGAUAUGGAACACAAGG	Knockdown
si*ADGRF5*-2	GACAGACCCUGUCAUAUAU	AUAUAUGACAGGGUCUGUC	Knockdown

Supplementary Table 2. Antibodies used in the study

Antibody	Company	Purposes
MMP8	Santa Cruz (sc-514803)	WB/IHC
β-ACTIN	Beyotime (AA128)	WB
RhoA	Santa Cruz (sc-418)	WB
Rac1	Sigma-Aldrich (05-389)	WB
Phospho-C/EBPβ (Thr235)	CST (#3084)	WB
C/EBPβ	CST (#43095)	WB/IF/ChIP
β-catenin	CST (#8480)	WB
ERK1/2	CST (##9102)	WB
Phospho-ERK1/2 (Thr202/Tyr204)	CST (#4370)	WB
FAK	CST (#3285)	WB
Phospho-FAK (Y397)	CST (#3283)	WB
Flag-tag	Sigma-Aldrich (F9291)	WB
α-Tubulin	Beyotime (AT819)	WB
GAPDH	Beyotime (AG019)	WB
goat anti-mouse IgG	Jackson	WB
goat anti-rabbit IgG	Jackson	WB
Src	CST (#2108)	IHC
Phospho-Src(Y416)	CST (#2101)	WB/IP
E-cadherin	CST (#3195)	WB
Phospho-SMAD2(Ser465/Ser467)	CST (#18338)	WB
SMAD2	CST (#8685)	WB
Histone 3	CST (#4499)	WB

Abbreviations: Western blotting (WB), Chromatin-immunoprecipitation assay (ChIP), Immunoprecipitation assay (IP), Immunohistochemistry assay (IHC).
