# Supplementary material for: The adhesion-GPCR ADGRF5 fuels breast cancer progression by suppressing the MMP8-mediated antitumorigenic effects
Source: Cell Death Dis. 2024 Jun 27;15(6):455. doi: 10.1038/s41419-024-06855-8 (PMC11211477; doi:10.1038/s41419-024-06855-8)

Figure 1F

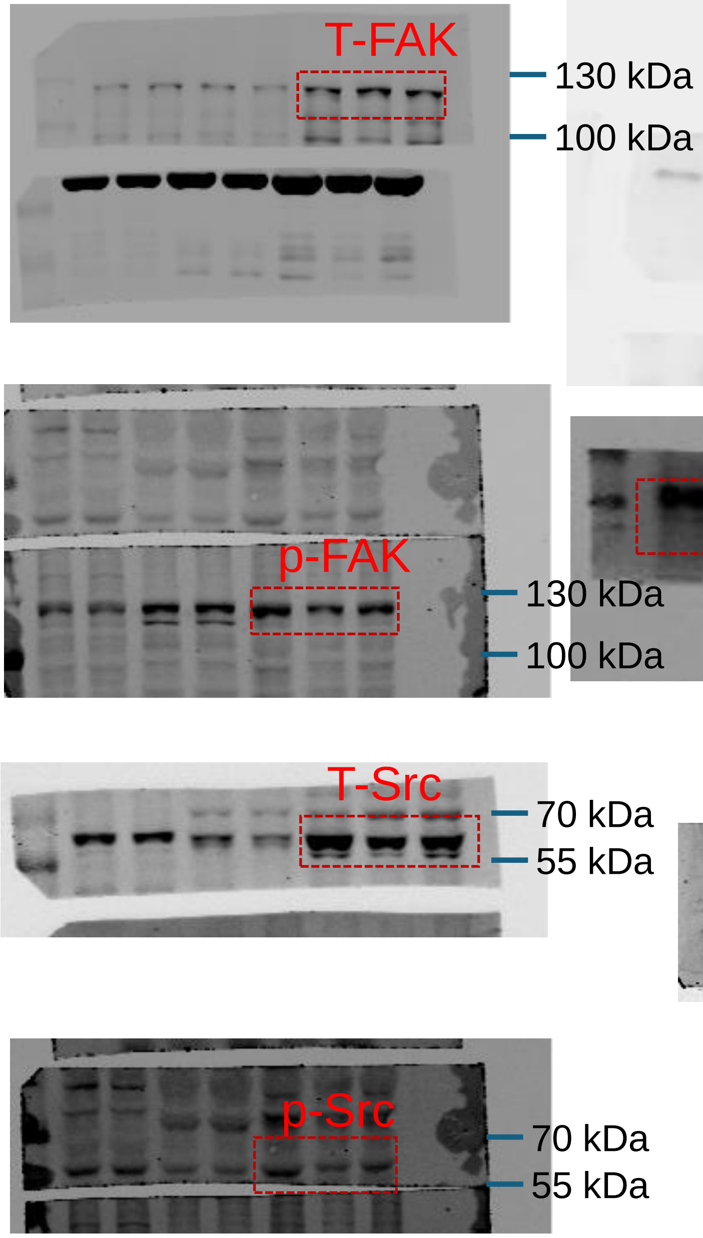

Figure 1H

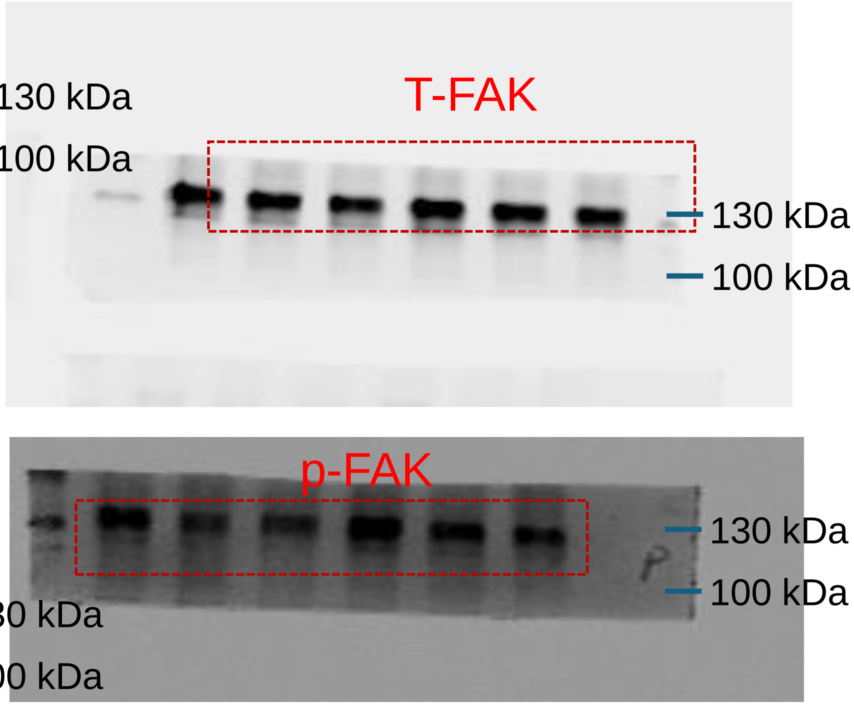

Figure 1J

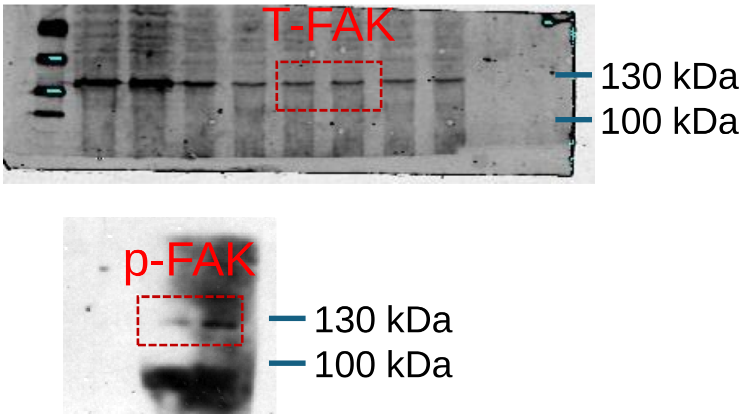

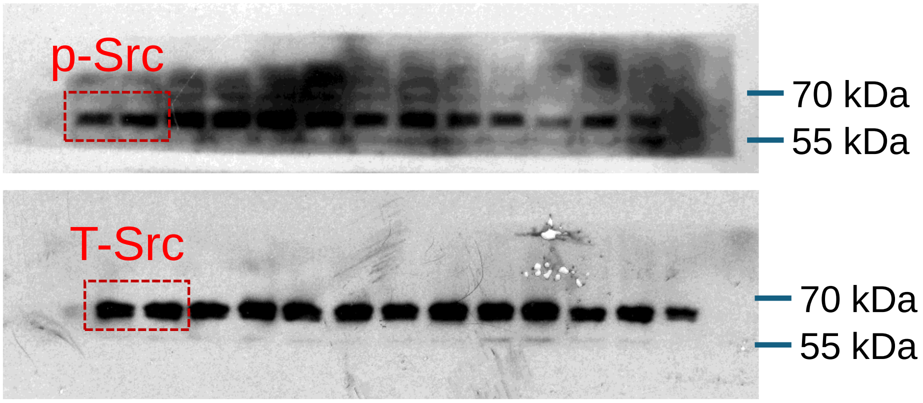

Figure 1L

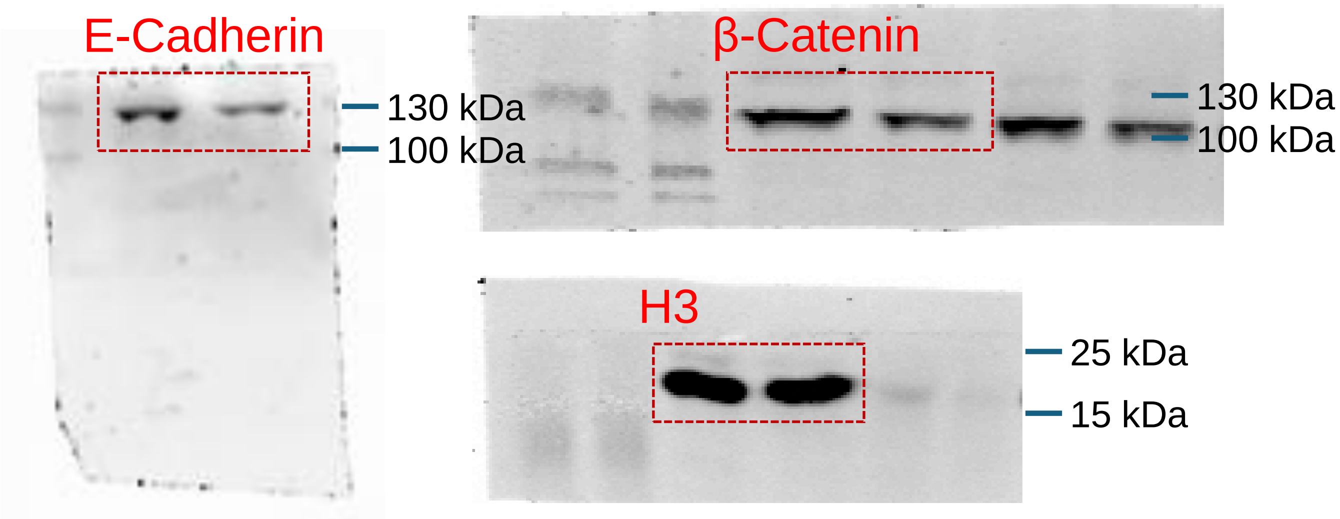

Figure 3C

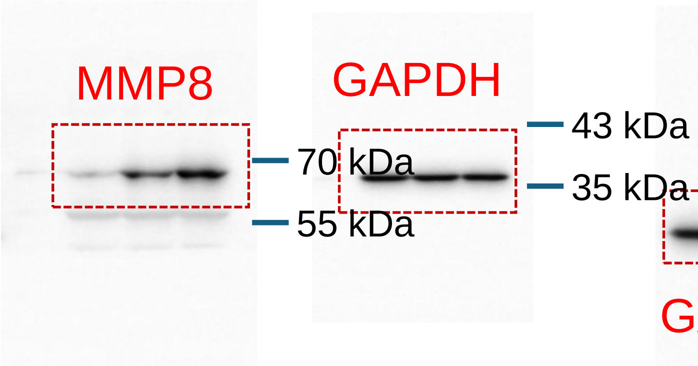

Figure 3D

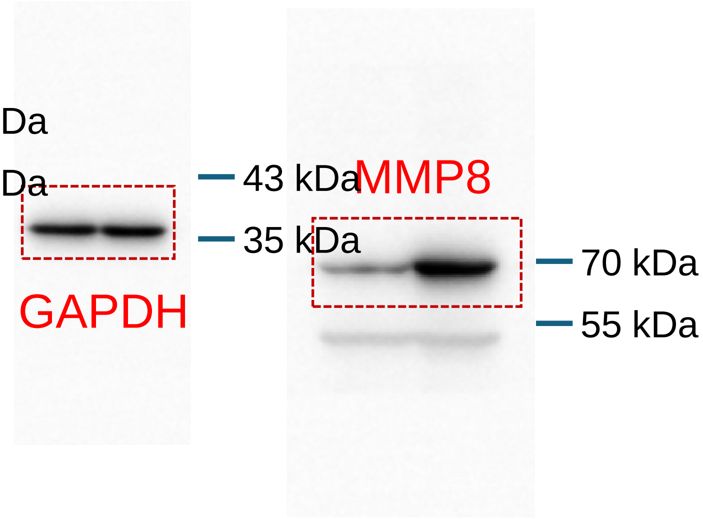

Figure 3E

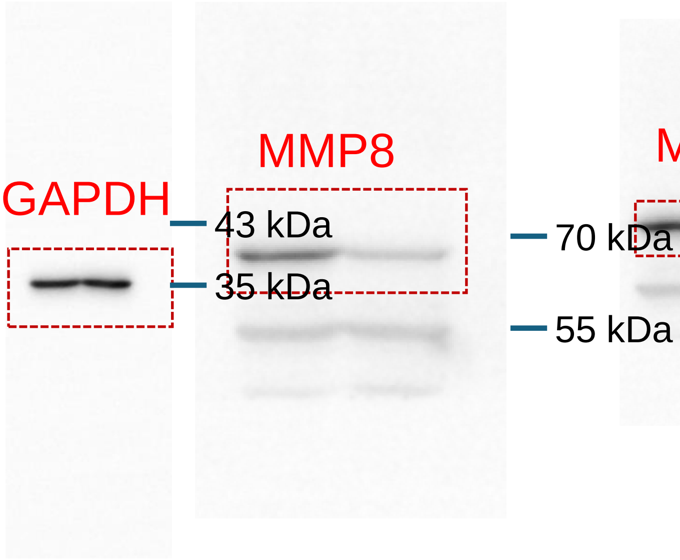

Figure 3F

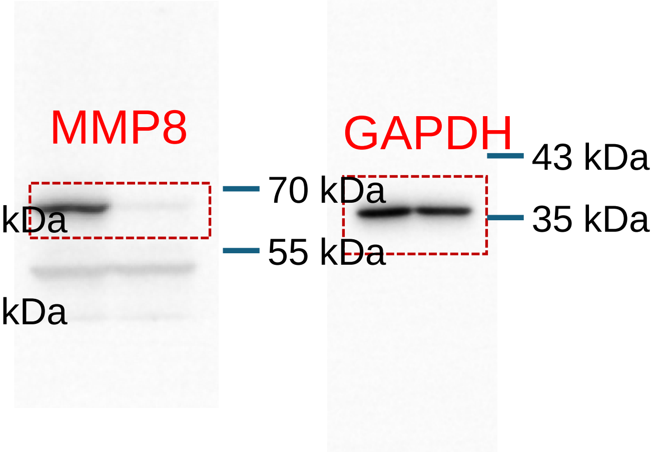

Figure 3I

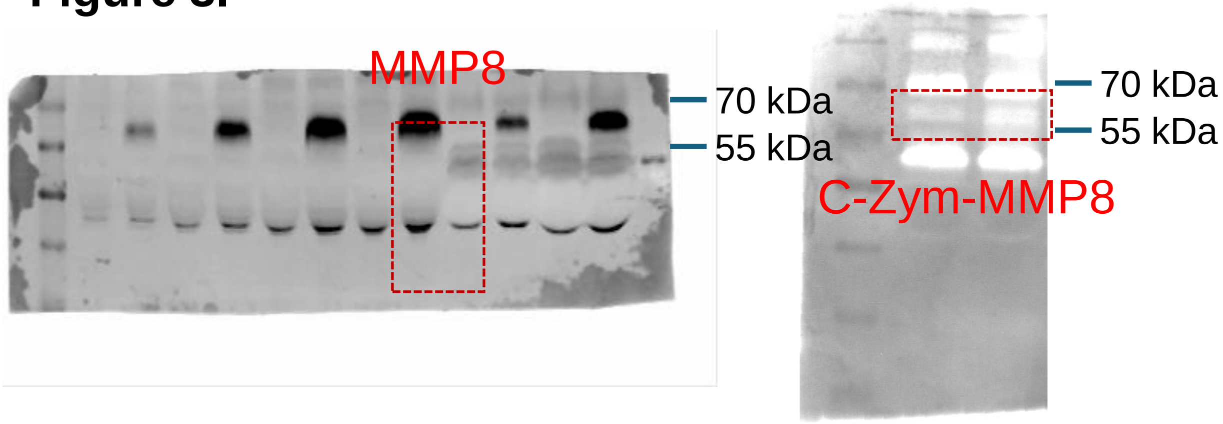

Figure 3L and M

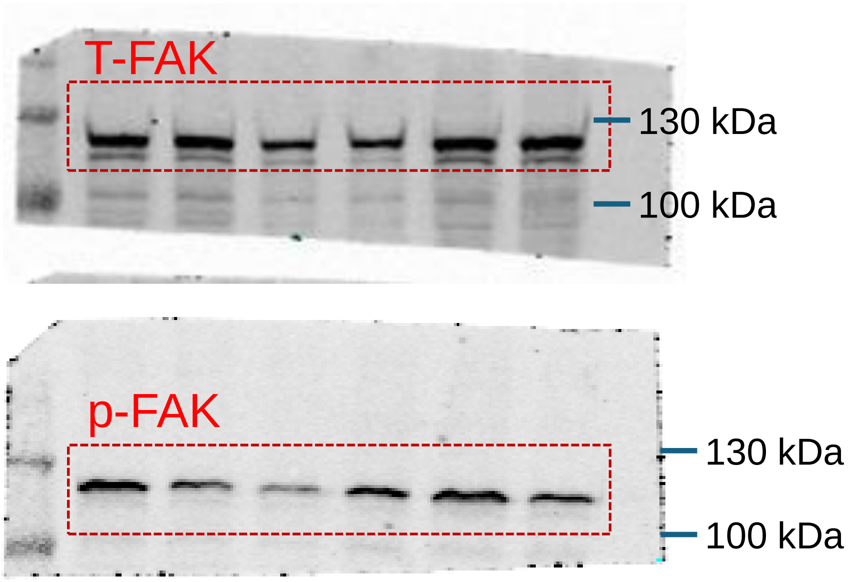

Figure 4E

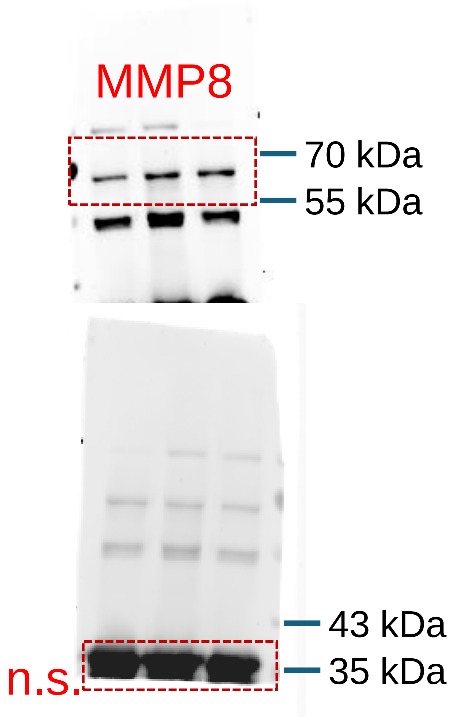

Figure 4G

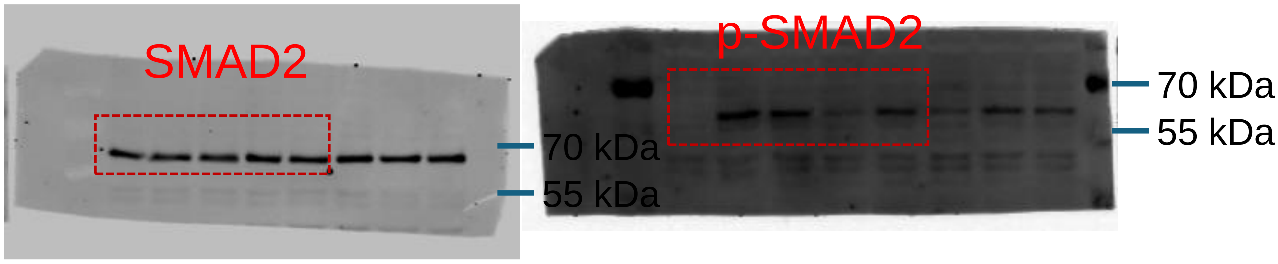

Figure 5E

Figure 5C

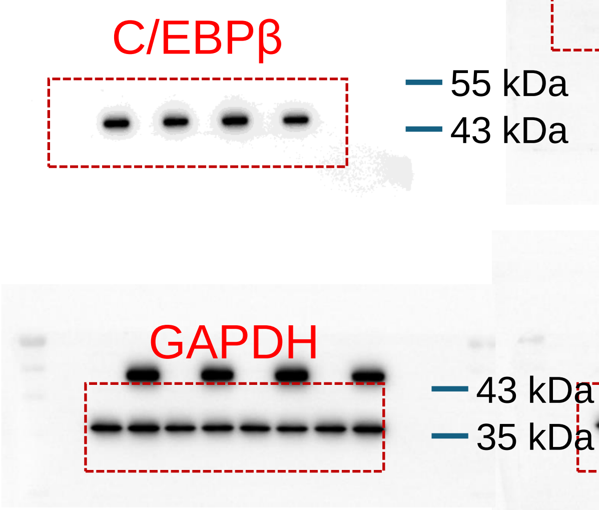

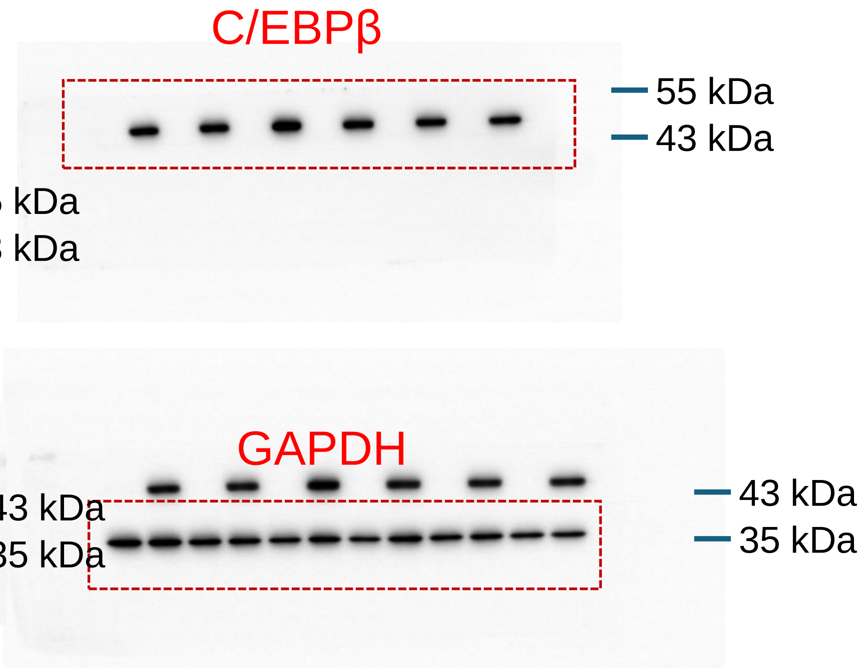

Figure 5F

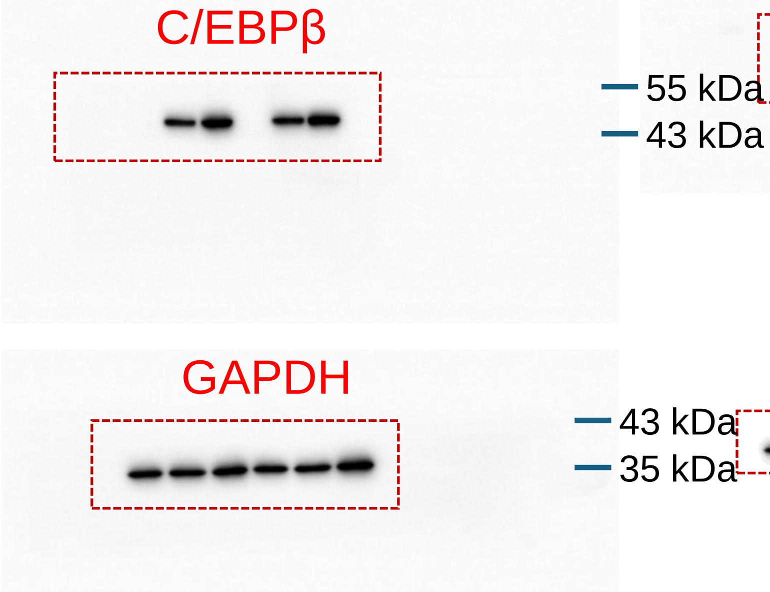

Figure 5G

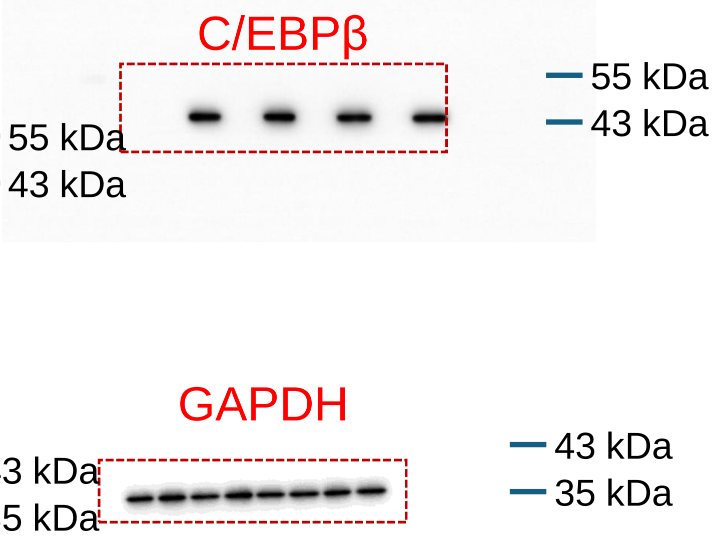

Figure 5I

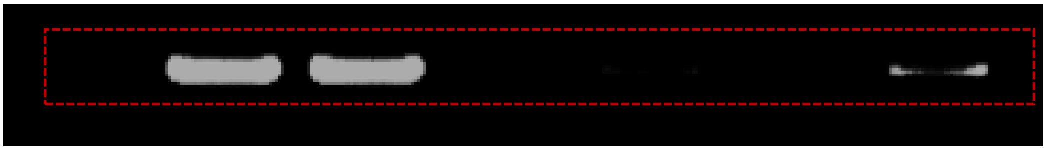

Figure 6B

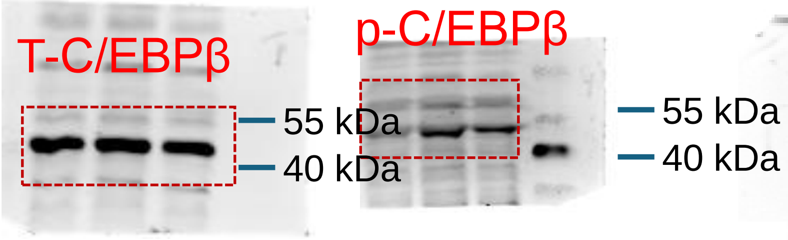

Figure 6E

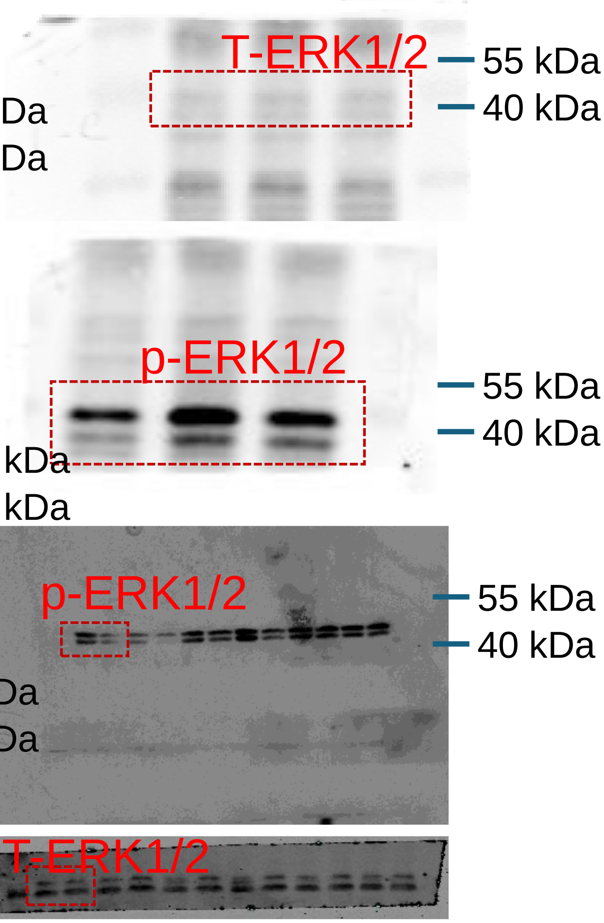

Figure 6I

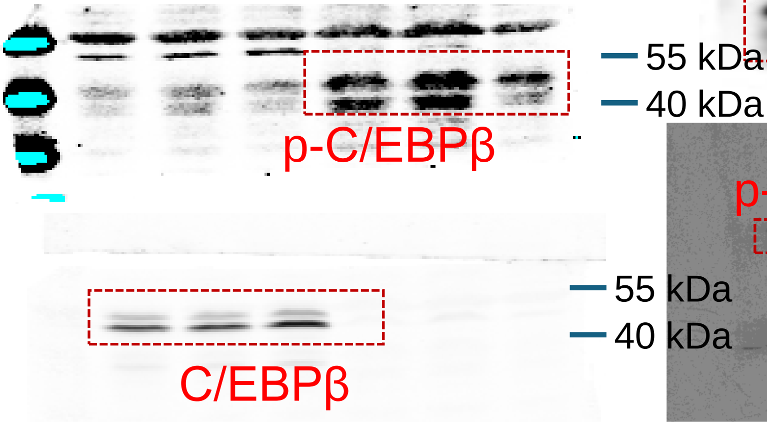

Figure 6K

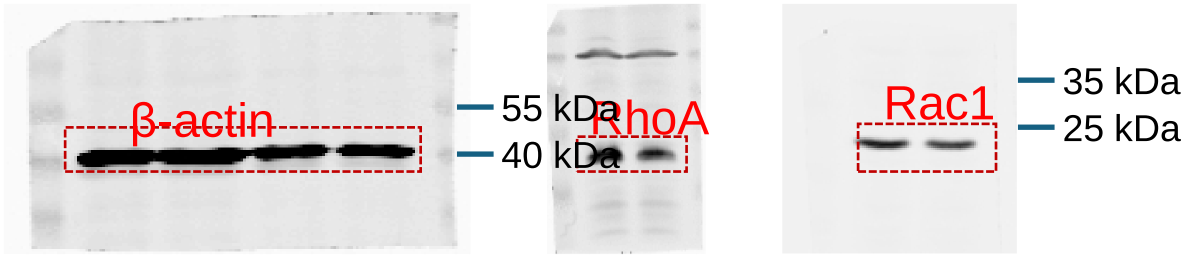

Figure 6M

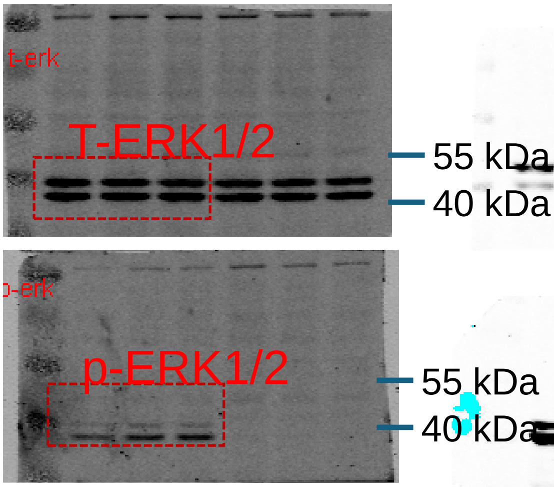

Figure 6Q

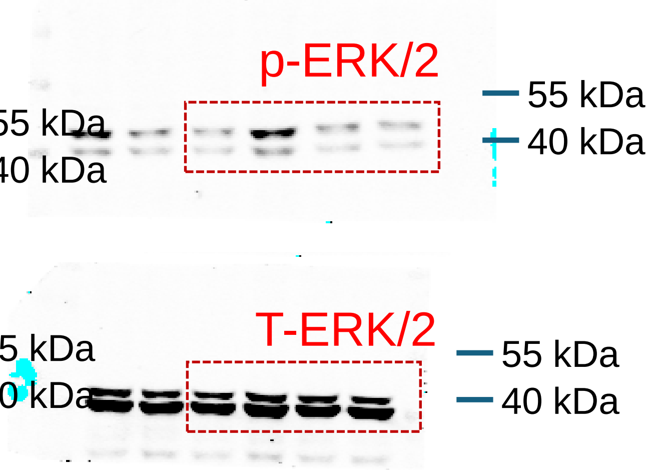

Supplement: Supplementary file 3 — Original western blots [file 41419_2024_6855_MOESM3_ESM.pdf]
